# Acute air pollution exposure and gastrointestinal cancer mortality: a case-crossover study in coastal China

**DOI:** 10.3389/fpubh.2025.1666928

**Published:** 2025-09-25

**Authors:** Zhuchao Wu, Chaohua Wei, Jian Sun, Beibei Qiu, Weimin Kong, Yang Yang, Yanqiu Huang, Cheng Li, Lingling Wu, Fudong Liu, Xiaojie Wang

**Affiliations:** ^1^School of Public Health and Management, Jiangsu Medical College, Yancheng, China; ^2^Department of Epidemiology, School of Public Health, Nanjing Medical University, Nanjing, China; ^3^Department of Thoracic Surgery, The First People’s Hospital of Yancheng City, The Yancheng Clinical College of Xuzhou Medical University, Yancheng, China; ^4^Department of Chronic Communicable Disease, Nanjing Municipal Center for Disease Control and Prevention, Nanjing, China; ^5^Department of Endocrinology, The First People’s Hospital of Yancheng City, The Yancheng Clinical College of Xuzhou Medical University, Yancheng, China; ^6^Bloomberg School of Public Health, Johns Hopkins University, Baltimore, MD, United States; ^7^State Key Laboratory of Systems Medicine for Cancer, Center for Single-Cell Omics, School of Public Health, Shanghai Jiao Tong University School of Medicine, Shanghai, China; ^8^Department of Epidemiology, School of Public Health, Cheeloo College of Medicine, Shandong University, Jinan, China; ^9^Department of Chronic and Non-Communicable Disease, Yancheng Municipal Center for Disease Control and Prevention, Yancheng, China; ^10^Department of Pharmacy, The Affiliated Hospital of Xuzhou Medical University, Xuzhou, China

**Keywords:** ambient particulate matter, gastrointestinal cancer, mortality risk, distributed lag nonlinear model, case-crossover study

## Abstract

**Background:**

Gastrointestinal (GI) cancers account for 43.1% of cancer-related deaths in China, with aging populations exacerbating this burden. While chronic air pollution exposure is linked to GI carcinogenesis, evidence on acute effects remains limited. This study investigates short-term ambient pollutant exposure and GI cancer mortality in a coastal Chinese city with moderate pollution levels.

**Methods:**

Using death registry data from Yancheng, China (2013–2022; *n* = 104,216 GI cancer deaths), we employed a time-stratified case-crossover design combined with distributed lag nonlinear models (DLNM) to assess associations between daily PM_2.5_, PM_10_, SO_2_, NO_2_, and O_3_ concentrations (lag 0–7 days) and mortality. Stratified analyses by age, sex, and cancer subsite were conducted, with sensitivity analyses evaluating model robustness.

**Results:**

A 10 μg/m^3^ increase in PM_2.5_, PM_10_, and O_3_ was associated with acute GI cancer mortality, peaking at lag 0–5 days (relative risk [RR] = 1.011, 95% CI: 1.000–1.022 for PM_2.5_; RR = 1.009, 95% CI: 1.001–1.017 for PM_10_; RR = 1.008, 95% CI: 1.001–1.016 for O_3_). The older males (≥65 years) exhibited heightened vulnerability, with maximal cumulative RRs of 1.018 (PM_2.5_), 1.010 (PM_10_), and 1.014 (O_3_). Esophageal cancer showed acute PM sensitivity (lag 0–4 days: RR = 1.021 for PM_2.5_), while colorectal cancer mortality correlated with delayed O_3_ effects (lag 0–7 days: RR = 1.031). No associations were observed for SO_2_ or NO_2_. Sensitivity analyses confirmed model stability across pollutant co-exposure adjustments and temporal confounders.

**Conclusion:**

Short-term exposure to PM_2.5,_ PM_10_, and O_3_ elevates GI cancer mortality risk, particularly among the older males and upper GI malignancies. These findings highlight the need for revised air quality standards addressing acute exposure thresholds and targeted protections for high-risk populations to mitigate pollution-related cancer mortality.

## 1 Introduction

Gastrointestinal (GI) cancer presents a significant global public health challenge. According to the 2022 cancer mortality estimates from the Global Cancer Observatory (GLOBOCAN), GI cancers at specific sites constitute five of the top 10 causes of cancer mortality. These include colorectal, liver, stomach, pancreatic, and esophageal cancers, accounting for 9.3%, 7.8%, 6.8%, 4.8% and 4.6% of total cancer deaths, respectively (1). China is expected to have 1.6 million new cases and 1.11 million deaths from digestive system cancers, representing 43.1% of all cancer-related deaths in 2022, with older people most affected (2). With the advent of China’s aging population, the burden of the expected future gastrointestinal cancer mortality and morbidity will increase.

Mounting epidemiological evidence positions ambient pollutants as critical modulators of carcinogenesis and tumor evolution, with their multifaceted biological mechanisms now constituting a priority research domain in environmental oncology. Environmental air pollution encompasses various contaminants including gaseous pollutants like sulfur dioxide (SO₂), nitrogen dioxide (NO₂), ozone (O_3_), and volatile organic compounds (VOCs) along with particulate matter (PM) (3). These pollutants often occur together, posing health risks to the population, particularly the harm of PM. The International Agency for Research on Cancer (IARC) has classified PM as a human carcinogen (4). Previous research has demonstrated a robust correlation between the short-term effects of air pollution on all-cause deaths and deaths from cardiovascular and respiratory diseases (5–8). Epidemiological literature demonstrates chronic particulate matter exposure exhibits dose–response relationships with both total and site-specific gastrointestinal cancer mortality (9, 10). A meta-analysis of 20 cohort studies quantified this association, revealing that 80% (16/20) of included research confirmed statistically significant associations between prolonged PM_2.5_/PM_10_ exposure and elevated GI cancer risk (11). Notably, current evidence exhibits three critical limitations: (1) paucity of investigations on acute (<7 days) pollution exposures’ impacts on digestive tract cancer outcomes; (2) absence of population-level studies in regions with ambient pollutant concentrations below WHO thresholds (e.g., Yancheng, China); (3) insufficient mechanistic exploration of particulate-induced gastrointestinal carcinogenesis. These knowledge gaps underscore the imperative for methodologically standardized investigations addressing geospatial heterogeneity in pollution exposure-response dynamics.

In this study, we analyzed death registry data from Yancheng City, China for the years 2013–2022. The dataset included over 100,000 deaths related to GI cancer. Our analytic framework combined a case-time-control design with distributed lag nonlinear modeling (DLNM) within a quasi-Poisson generalized additive model architecture to quantify concentration-response relationships between acute ambient pollutant exposures (lag 0–7 days) and gastrointestinal cancer mortality outcomes. Furthermore, we conducted stratified analyses by sex and age to explore the potential moderating effects and identify potentially susceptible populations. These findings not only helped identify high-risk susceptible populations but also provided a crucial epidemiological foundation for the development of effective preventive measures against GI cancer.

## 2 Materials and methods

### 2.1 Study area and population

Yancheng is a coastal city situated in the eastern part of China, within the transitional belt from the subtropical to warm temperate zones. It serves as a pivotal link between northern and southern China, encompassing an area of 17,718 square kilometers with a population of 6,689,700 in 2022. The average annual temperature is recorded at 16.1 °C. Despite a decreasing trend over the past decade, PM_2.5_ levels in Yancheng persistently exceed the World Health Organization (WHO) air quality guideline (annual mean of 5 μg/m^3^) by a substantial margin.

### 2.2 Daily mortality data

This study analyzed GI cancer mortality patterns using de-identified records from Yancheng, China, spanning 2013–2022. Mortality data were systematically collected through the municipal Death Registration System under the supervision of Yancheng Municipal Center for Disease Control and Prevention (CDC)., covering all residential areas within the jurisdiction. GI cancer cases were specifically identified using ICD-10 codes C15-C26. To ensure data reliability, the CDC implemented multilevel quality control protocols, including routine audits and validation processes for all reported deaths. The surveillance framework adheres to standardized national procedures for cause-of-death certification and coding practices.

### 2.3 Daily air quality and meteorological data

We extracted data on meteorological factors (temperature, relative humidity, wind speed, barometric pressure) in Yancheng between January 1, 2013 and December 31, 2022 from the China Meteorological Data Sharing Center^1^, as well as daily average concentrations of five ambient air pollutants, including PM_10_, PM_2.5_, SO_2_, NO_2_, and O_3_ (the concentration of O_3_ was the maximum 8-h moving average) in Yancheng during the same period from the National urban air quality real-time release platform^2^. Air-pollution data obtained from this monitoring system have been used extensively to evaluate the health effects of air pollution both regionally and nationally (12, 13). The 2013–2022 timeframe was selected because 2013 marked a pivotal year for air quality monitoring in China: the national ambient air monitoring network was officially operationalized on January 1, 2013. This network enabled 74 key cities (including Yancheng) to commence standardized monitoring and real-time public disclosure of the 6 pollutants (including PM_2.5_) and AQI indices, ensuring high-quality, consistent data from 2013 onward. The monitoring sites in Yancheng were deployed following the Technical Specifications for Ambient Air Quality Monitoring Network (HJ 664–2013), ensuring effective representation of spatial variations in pollutant concentrations.

### 2.4 Statistical analysis

This study adopted a time-stratified case-crossover design to control for both known (e.g., age, socioeconomic status, sex) and unknown confounding factors by matching each death case to its exposure status across different time periods (14, 15). Specifically, the stratification variable “year-month-day of the week” was used to select control periods (i.e., dates with the same year, month, and day of the week) for each case, thereby eliminating interference from long-term trends, seasonal variations, and weekday-related effects. Building on this, a quasi-Poisson regression model was employed to analyze the stratified data. This approach not only addresses overdispersion and autocorrelation in daily GI cancer death counts but also ensures robustness by conditionally adjusting for stratification variables through a fixed-effects framework (16, 17). To further capture delayed and nonlinear effects of environmental exposures, the study integrated a Distributed Lag Non-linear Model (DLNM) to quantify the dynamic impacts of exposure factors (e.g., air pollutants) across varying lag periods (e.g., 0–7 days post-exposure) and to dissect their nonlinear relationships with mortality risk. By combining these methods, the model leverages the self-matching advantages of the case-crossover design to control for confounders while utilizing the DLNM to flexibly model complex temporal exposure-response patterns. This integration enables a more precise assessment of the acute effects of environmental factors on gastrointestinal cancer mortality (14, 15).

Specifically, the model uses the expected number of deaths due to GI cancer on observation day *t*, denoted as *E(Y_t_)*, as the dependent variable. The log-linear formulation is expressed as:

*Y_t~_ quasi-Poisson[E(Y_t_*)*]*

Log[E(Y_t_)] = α + βP_t,l_ + γT_t,l_ + ns(Relative Humidity_t_, df = 3) + ns(Wind Speed_t_, df = 3) + ns(time,7*10) + stratum + dow + as factor(holiday)

*P_t,l_* and *T_t,l_* represent cross-basis functions of daily average concentration for air pollutants and daily mean temperature, respectively, which quantify the exposure-response relationships of pollutants and temperature across varying lag days; The coefficients *β* and *γ* correspond to these functions, respectively; Natural cubic splines (*ns*) with 3 degrees of freedom (df) are employed to control for nonlinear confounding effects of *relative humidity* and *wind speed* (18). The stratification variable (stratum) inherently adjusts for seasonality and long-term trends by restricting comparisons to days within the same year, month, and day of the week. The time-stratified case-crossover approach inherently adjusts for temporal confounders including weekly variations, seasonal cycles, and longitudinal trends through its self-matching design framework. Furthermore, to refine control over short-term temporal variations, the model incorporates day-of-week effects (dow) to capture mortality fluctuations linked to differences between weekdays and weekends, and a categorical holiday variable to account for public holidays. In the analysis of lagged exposure effects, the study evaluates both single-day lags (lag0–lag7, corresponding to the exposure day up to 7 days prior) and cumulative lags (lag01–lag07, defined as moving averages of the current day and the preceding 1–7 days) to comprehensively assess the acute health impacts of pollutants (19).

Single-pollutant models were established for PM_2.5_, PM_10_, SO_2_, NO_2_, and O_3_, independently assessing the lag-specific and cumulative effects of each pollutant on GI cancer mortality per 10-unit increase in concentration. Subsequently, to address the collinearity between air pollutants, multi-pollutant models were constructed by excluding highly correlated variables (Pearson correlation coefficient *r* < 0.8) based on statistically significant pollutants identified in single-pollutant analyses. Relative risk (RR) estimates and corresponding 95% confidence intervals (CI) were employed to represent the lag-specific and cumulative exposure impacts. Further, stratified analyses by age (<65 years vs. ≥65 years), and sex (male vs. female) were conducted to reveal population heterogeneity in risk distribution and identify potential high-risk subpopulations.

We finally performed several sensitivity analyses to evaluate the robustness of the results. Firstly, to evaluate model parameter sensitivity, we systematically varied the df for the time variable in single-pollutant models, testing a range of 6–10 df per year as supported by prior methodological studies (20, 21). Secondly, to address multicollinearity concerns, dual-pollutant models were implemented using conditional inclusion criteria (Spearman’s *ρ* < 0.7) to quantify confounding interactions between co-varying atmospheric contaminants. Thirdly, recognizing the unprecedented environmental and healthcare disruptions caused by the SARS-CoV-2 pandemic, we introduced binary dummy variables (2020–2021 coded as 1 vs. other years as 0) into our primary models to isolate and control for pandemic-related confounding factors.

Analytical procedures were implemented in R version 4.2.2 (R Foundation for Statistical Computing) with specialized computational libraries: the ‘dlnm’ package for distributed lag nonlinear modeling, and ‘splines’ for nonparametric smoothing. A two-tailed alpha level of 0.05 served as the prespecified statistical significance threshold.

### 2.5 Ethics approval

Ethics approval was not required for secondary analysis of the anonymous data in this study.

## 3 Results

### 3.1 Descriptive statistics

As shown in Table 1, there were 104,216 GI cancer deaths identified in Yancheng from 2013 to 2022 averaging 28.62 deaths per day. The majority of deaths died from esophagus cancer (29.94%), stomach cancer (28.11%), and liver cancer (22.14%), while the number of colorectum cancer (10.41%) and pancreas cancer (7.89%) deaths was relatively small. A total of 68,247(65.49%) males and 35,969 (34.51%) females were among all GI cancer deaths. The number of deaths per day attributed to GI cancer was 18.74 for males and 9.88 for females. In all cases of death caused by GI cancer, 71.21% of patients were aged 65 or older, whereas 28.79% were younger than 65 years old.

**Table 1:** Summary statistics for air pollutants, meteorological parameters and GI cancer daily deaths from January 2013 to December 2022 in Yancheng, China.

Variables	Mean	SD	Min	P25	Median	P75	Max	Total
Daily air pollutants, μg/m^3^								
SO_2_, μg/m^3^	12.61	11.11	2.00	6.00	9.00	16.00	102.00	–
NO_2_, μg/m^3^	23.89	13.34	3.00	14.00	20.00	30.00	100.00	–
O_3_, μg/m^3^	104.89	37.18	12.00	77.00	99.00	125.00	261.00	
PM_10_, μg/m^3^	74.58	51.77	0.00	40.00	60.00	95.00	588.00	–
PM_2.5_, μg/m^3^	44.03	38.36	0.00	19.00	33.00	56.00	392.00	–
Meteorological factors								
Temperature, °C	15.88	9.33	−9.10	7.70	16.40	23.90	34.70	–
Relative humidity, %	75.04	13.09	31.30	67.00	76.40	84.80	100.00	–
Pressure, Pa	1016.34	9.41	985.60	1008.20	1016.60	1023.80	1041.40	–
Wind speed, m/s	2.38	1.06	0.30	1.60	2.20	3.00	8.10	–
GI Cancer daily deaths, *n*								
Total daily deaths	28.62	5.75	11	25	28	32	55	104,216
Esophagus cancer	8.57	3.02	0	6	8	10	21	31,198
Stomach cancer	8.04	2.95	1	6	8	10	21	29,298
Liver cancer	6.08	2.58	0	4	6	8	17	22,136
Colorectum cancer	2.98	1.83	0	2	3	4	12	10,846
Pancreas cancer	2.26	1.56	0	1	2	3	11	8,220
Gender								
Male, *n*	18.74	4.59	6	16	18	22	41	68,247
Female, *n*	9.88	3.22	0	8	10	12	23	35,969
Age								
≥65 years	20.38	5.00	6	17	20	24	39	74,214
<65 years	8.24	3.19	0	6	8	10	21	30,002
Warm season	28.32	5.65	11	25	28	32	55	51,832
Cold season	28.91	5.82	14	25	29	33	51	52,384

The season was divided into warm (April to September) and cold (October to March).

NO_2_, nitrogen dioxide; O_3,_ ozone; PM_10_, particulate matter with an aerodynamic diameter <10 mm; SO_2_, sulfur dioxide.

**Figure 1:**
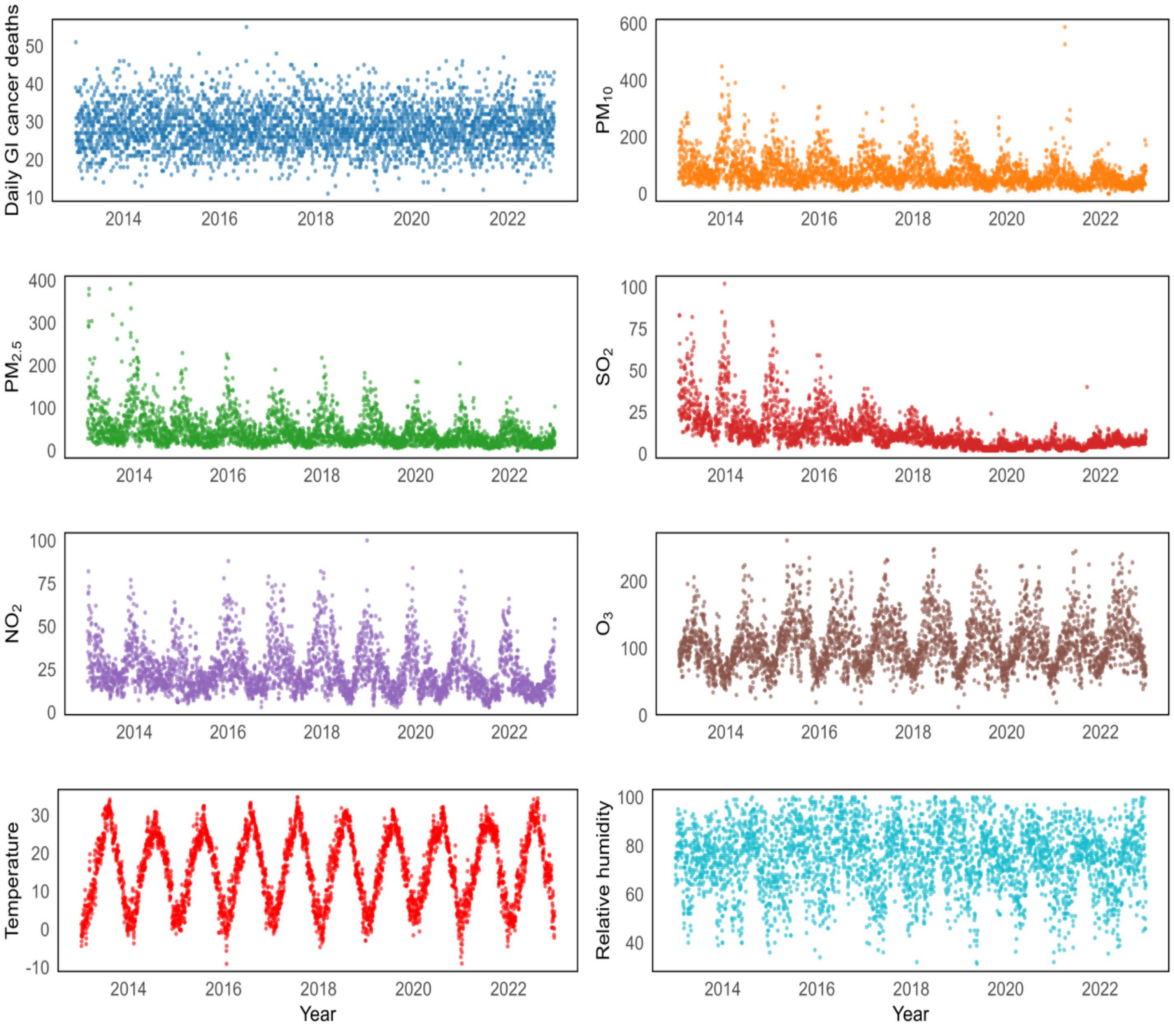
Temporal patterns of daily GI cancer deaths and various environmental factors from 2013 to 2022.

The daily average concentrations of SO_2_, NO_2_, O_3_, PM_10_ and PM_2.5_ in Yancheng were 12.61 μg/m^3^, 23.89 μg/m^3^, 104.89 μg/m^3^, 74.58 μg/m^3^ and 44.03 μg/m^3^, respectively. During the study period, the daily average temperature was 15.88 °C, the mean pressure was 1016.34 kPa, the mean wind speed was 2.38 m/s, and the relative humidity was 75.04%. The time-series patterns of ambient air pollution, daily GI cancer deaths, and meteorological factors between 2013 and 2022 are shown in Figure 1.

### 3.2 Spearman rank correlation analysis

Supplementary Table S1 presents Pearson correlation coefficients among air pollutant concentrations. Significant inter-correlations were observed (*p* < 0.001) among sulfur dioxide (SO₂), nitrogen dioxide (NO₂), and particulate matter (PM_10_ and PM_2.5_), while ozone (O_3_) demonstrated associations exclusively with PM_10_ and NO₂ in the analysis. Our research results indicated that temperature exhibits a positive correlation with O₃ concentration, yet a negative correlation with PM_2.5_, PM_10_, SO₂, and NO₂ concentrations (all *p* < 0.001). Relative humidity also demonstrated a negative correlation with these five pollutant concentrations (all *p* < 0.001). Furthermore, atmospheric pressure was positively correlated with PM_2.5_, PM_10_, SO₂, and NO₂ concentrations, and negatively correlated with O₃ concentration (all *p* < 0.001). Wind speed exhibited a negative correlation with PM_2.5_, PM_10_, O₃, and NO₂ concentrations, but a positive correlation with SO₂ concentration (all *p* < 0.01).

### 3.3 The short-term exposure to air pollution and GI cancer deaths

**Figure 2:**
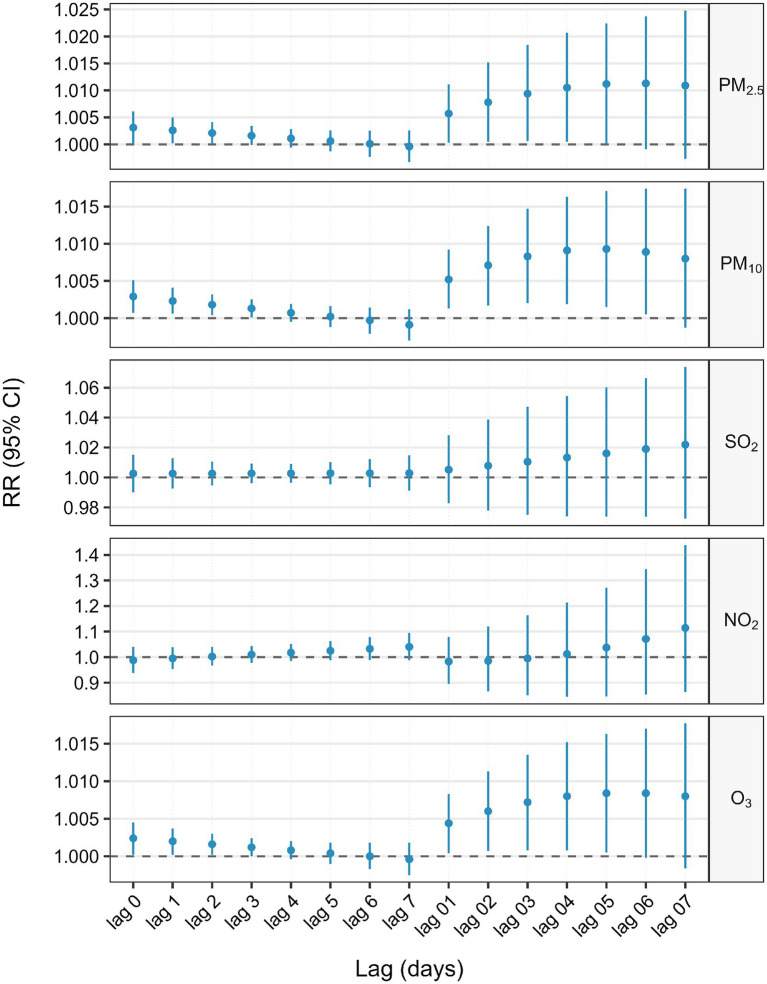
Association between 10 μg/m^3^ air pollution exposure and lagged GI cancer mortality risk: single-pollutant model.

Figure 2 and Table 2 estimated lag-response and cumulative relative risk of GI cancer deaths associated with a 10 μg/m^3^ increase in air pollutant concentrations using a single pollutant model. For single–day lags, significant positive associations were found between these pollutants (PM_2.5_ and O_3_) and GI cancer deaths at lag 0, lag 1 and lag 2 (lag 0, lag 1, lag 2 and lag 3 days for PM_10_), with all these pollutants peaking on lag 0 day. For multi-day lags, significant positive associations were also found between these pollutants (PM_2.5_, PM_10_, and O_3_) and GI cancer deaths. The cumulative risk of GI cancer deaths was associated with PM_2.5_ exposure, ranging from lag 0 day (RR = 1.0031, 95%CI: 1.0001–1.0061) to lag 0–5 days (RR = 1.0112, 95%CI: 1.0001–1.0224). PM_10_ exposure, from lag 0 day (RR = 1.0029, 95%CI: 1.0007–1.0051) to lag 0–6 days (RR = 1.0089, 95%CI: 1.0005–1.0174), was linked to an increased cumulative risk of GI cancer deaths. O_3_ exposure, from lag 0 days (RR = 1.0024, 95%CI: 1.0002–1.0045) to lag 0–5 days (RR = 1.0084, 95%CI: 1.0005–1.0163), also increased the cumulative risk of GI cancer deaths. Moreover, the estimated cumulative relative risk of GI cancer deaths associated with a 10 μg/m^3^ increment in pollutant concentrations all reached a maximum at lag 0–5 days for PM_2.5_, PM_10_, and O_3_. There was no single-day or multi-day lag effect on SO_2_ and NO_2_.

**Table 2:** Association between 10 μg/m^3^ air pollution exposure and lagged GI cancer mortality risk: single-pollutant model.

Lag days	PM_2.5_ (RR [95%CI])	PM_10_ (RR [95%CI])	SO_2_ (RR [95%CI])	NO_2_ (RR [95%CI])	O_3_ (RR [95%CI])
Lag 0	**1.0031(1.0001,1.0061)**	**1.0029(1.0007,1.0051)**	1.0026(0.9901,1.0152)	0.9877(0.9384,1.0395)	**1.0024(1.0002,1.0045)**
Lag 1	**1.0026(1.0002,1.0050)**	**1.0023(1.0006,1.0041)**	1.0026(0.9926,1.0127)	0.9950(0.9533,1.0385)	**1.0020(1.0002,1.0037)**
Lag 2	**1.0021(1.0001,1.0041)**	**1.0018(1.0004,1.0032)**	1.0026(0.9947,1.0106)	1.0024(0.9668,1.0394)	**1.0016(1.0002,1.0030)**
Lag 3	1.0016(0.9999,1.0034)	**1.0013(1.0001,1.0025)**	1.0027(0.9962,1.0092)	1.0099(0.9777,1.0431)	1.0012(1.0000,1.0024)
Lag 4	1.0011(0.9994,1.0028)	1.0007(0.9995,1.0019)	1.0027(0.9965,1.0090)	1.0174(0.9849,1.0509)	1.0008(0.9996,1.0020)
Lag 5	1.0006(0.9987,1.0026)	1.0002(0.9988,1.0016)	1.0028(0.9954,1.0102)	1.0249(0.9884,1.0627)	1.0004(0.9990,1.0018)
Lag 6	1.0001(0.9977,1.0025)	0.9997(0.9979,1.0014)	1.0028(0.9935,1.0122)	1.0325(0.9892,1.0777)	1.0000(0.9983,1.0018)
Lag 7	0.9996(0.9967,1.0026)	0.9991(0.9970,1.0012)	1.0029(0.9912,1.0147)	1.0402(0.9883,1.0948)	0.9996(0.9975,1.0018)
Lag 01	**1.0057(1.0003,1.0111)**	**1.0052(1.0013,1.0092)**	1.0052(0.9827,1.0281)	0.9828(0.8949,1.0792)	**1.0044(1.0004,1.0083)**
Lag 02	**1.0078(1.0005,1.0152)**	**1.0071(1.0017,1.0124)**	1.0078(0.9779,1.0387)	0.9851(0.8664,1.1201)	**1.0060(1.0007,1.0113)**
Lag 03	**1.0094(1.0006,1.0184)**	**1.0083(1.0020,1.0147)**	1.0105(0.9751,1.0473)	0.9949(0.8504,1.1639)	**1.0072(1.0008,1.0135)**
Lag 04	**1.0105(1.0005,1.0207)**	**1.0091(1.0019,1.0163)**	1.0133(0.9739,1.0543)	1.0121(0.8446,1.2129)	**1.0080(1.0008,1.0152)**
Lag 05	**1.0112(1.0001,1.0224)**	**1.0093(1.0015,1.0171)**	1.0161(0.9738,1.0602)	1.0373(0.8466,1.2711)	**1.0084(1.0005,1.0163)**
Lag 06	1.0113(0.9991,1.0237)	**1.0089(1.0005,1.0174)**	1.0190(0.9738,1.0663)	1.0711(0.8538,1.3437)	1.0084(0.9998,1.0170)
Lag 07	1.0109(0.9973,1.0248)	1.0080(0.9987,1.0174)	1.0219(0.9725,1.0738)	1.1141(0.8632,1.4380)	1.0080(0.9984,1.0177)

The bold number indicates the *P* < 0.05.

RR, relative ratio.

NO_2_, nitrogen dioxide; O_3,_ ozone; PM_10_, particulate matter with an aerodynamic diameter <10 mm; SO_2_, sulfur dioxide.

### 3.4 Subgroup analysis

**Figure 3:**
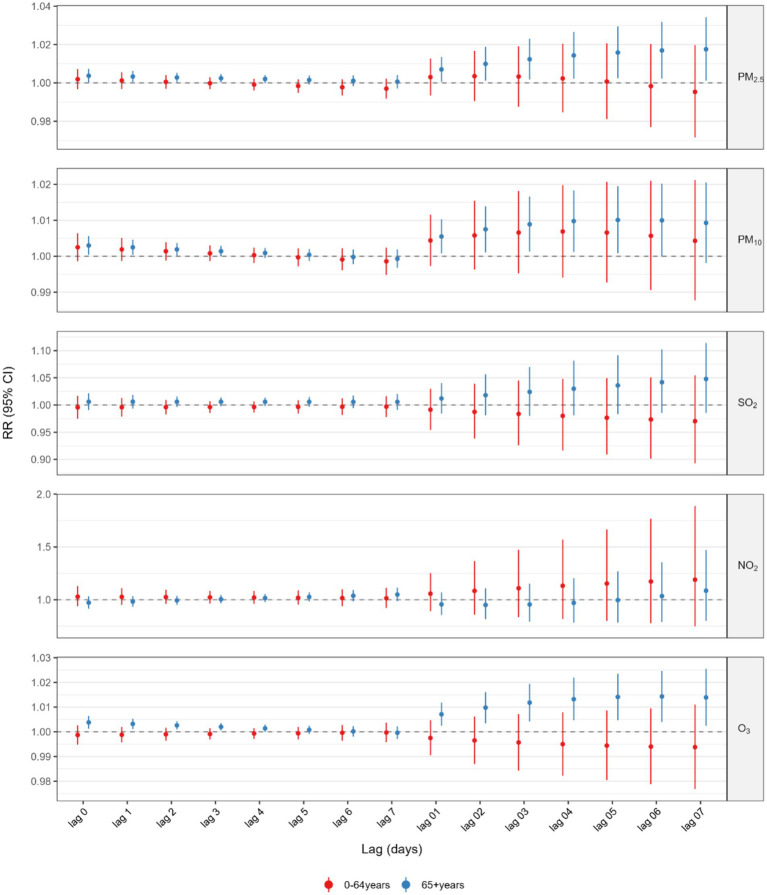
Association between 10 μg/m^3^ air pollution exposure and lagged GI cancer mortality risk: age-satisfied analysis.

**Figure 4:**
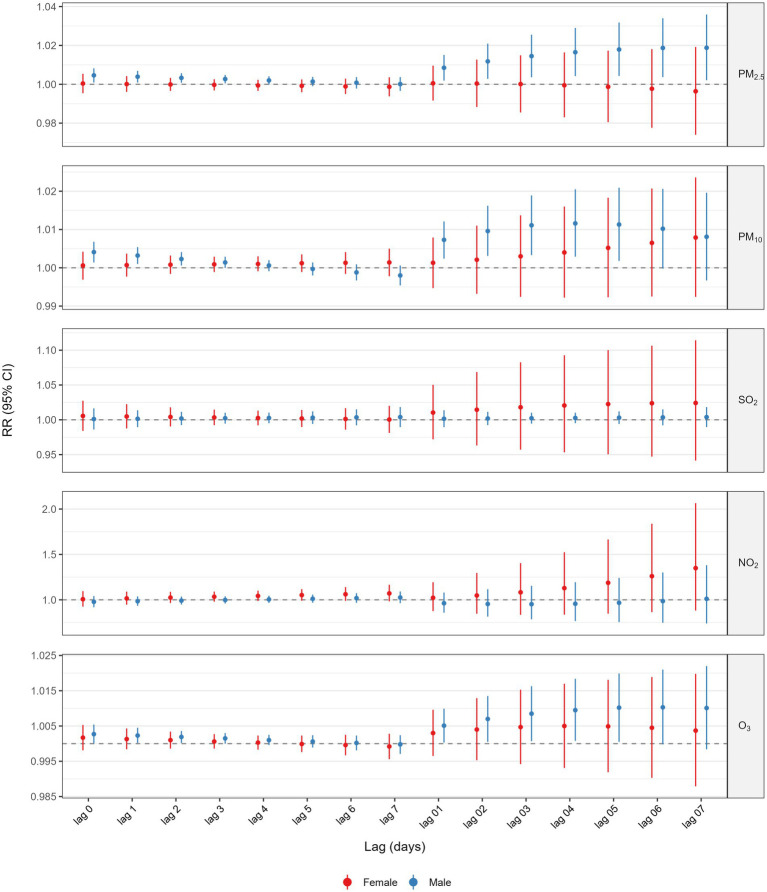
Association between 10 μg/m^3^ air pollution exposure and lagged GI cancer mortality risk: sex-stratified analysis.

Figure 3 and Supplementary Tables S2, S3 presented the results of the analysis stratified by various age groups within the single pollutant model. When categorized by age, the correlation between GI cancer deaths and exposure to PM_2.5_, PM_10_, and O_3_ was significant exclusively in the older population (age ≥65 years). The highest cumulative RR for PM_2.5_ was observed at a lag of 0–7 days (RR = 1.0175, 95%CI: 1.0011–1.0342), for PM_10_ at a lag of 0–5 days (RR = 1.0101, 95%CI: 1.0008–1.0195), and for O_3_ at a lag of 0–6 days (RR = 1.0143, 95%CI: 1.0040–1.0247). Additionally, we also performed a stratified analysis by gender (Figure 4 and Supplementary Tables S4, S5). We found that the statistically significant correlation between exposure to PM_2.5_, PM_10_, and O_3_ and GI cancer mortality was evident only in males. The association between PM_2.5_ exposure and GI cancer deaths was strongest at a lag of 0–7 days (RR = 1.0188, 95%CI: 1.0021–1.0359). For PM_10_, the peak cumulative risk was at a lag of 0–4 days (RR = 1.0116, 95%CI: 1.0029–1.0205), and the cumulative RR for O_3_ peaked at a lag of 0–5 days (RR = 1.0102, 95%CI: 1.0005–1.0199). No link with GI cancer mortality was identified for SO_2_ and NO_2_ in the subgroup analysis. Furthermore, our stratified analysis of five GI cancers revealed distinct anatomic and temporal susceptibility patterns to airborne pollutants (Supplementary Tables S6–S10). Esophageal cancer mortality exhibited multipollutant sensitivity, with PM_2.5_ demonstrating peak cumulative risk at lag 0–4 days (RR = 1.0216 per 10 μg/m^3^ increase; 95% CI: 1.0035–1.0401), concurrent with PM10’s maximal effect window during the same exposure period (RR = 1.0208, 95% CI: 1.0078–1.0340 per 10 μg/m^3^ increment). Notably, O_3_ displayed acute-phase toxicity in esophageal cancer, showing a significant mortality elevation at lag 0–2 days (RR = 1.0095, 95% CI: 1.0000–1.0192 per 10 μg/m^3^ increment). In contrast, colorectal cancer mortality was uniquely associated with ozone exposure, manifesting maximal risk at lag 0–7 days (RR = 1.0312, 95% CI: 1.0019–1.0614 per 10 μg/m^3^ increment). This divergence in temporal dynamics–early particulate matter dominance in upper gastrointestinal malignancies versus delayed ozone effects in lower gastrointestinal sites – was further reinforced by null associations observed in gastric, hepatic, and pancreatic cancers.

### 3.5 Sensitivity analysis

The robustness of the primary findings was systematically evaluated through multiple approaches. First, varying degrees of freedom (6–10 df per year) to adjust for long-term temporal trends yielded notably stable results across all exposure-pollutant models (Supplementary Tables S11–S14), demonstrating the consistency of effect estimates under different smoothing parameterizations. Furthermore, in two-pollutant models adjusting for potential confounding by co-pollutants, the associations between a 10 μg/m^3^ increase in PM_2.5_, PM_10_, SO_2_, NO_2_, and O_3_ concentrations and GI cancer mortality revealed no substantial differences compared to single-pollutant models (Supplementary Table S15). Additionally, the inclusion of dummy variables to account for the SARS-CoV-2 pandemic period revealed no substantial alterations in exposure-response relationships compared to the main model (Supplementary Table S16). Finally, we conducted additional sensitivity analyses examining the cumulative lag effect of air pollutants on GI cancer deaths over 0-21 days and found that the cumulative relative risks (RRs) for PM_2.5_, PM_10_, SO_2_, NO_2_, and O_3_ (per 10 μg/m^3^ increase) showed no evidence of decline over the 0–21 day period; instead, they remained stable or slightly increased (Supplementary Table S17).

## 4 Discussion

Our study reveals novel associations between short-term exposure to ambient pollutants and elevated GI cancer mortality in a coastal city with moderate pollution. Acute-phase risks peaked at lag 0–5 days (PM_2.5_, PM_10_, and O_3_), aligning mechanistically with pollutant-triggered systemic inflammation and oxidative stress, despite concentrations below WHO interim targets. These findings also demonstrated that transient pollution spikes may accelerate GI cancer mortality in vulnerable populations (e.g., the older population and men). Anatomic-temporal divergence—acute upper GI effects versus delayed lower GI impacts—highlights subsite-specific vulnerabilities, addressing prior ecological studies’ lack of subsite stratification.

The association between air pollutants and GI cancer mortality exhibits significant spatiotemporal heterogeneity. While evidence on the short-term effects of air pollution on GI cancer mortality remains limited, accumulating studies highlight significant associations with long-term exposure to air pollution on GI cancer mortality (22–26). Regarding the effects of PM_2.5_, discrepancies exist between our findings and previous short-term exposure studies: A Brazilian cohort demonstrated that each 10 μg/m^3^ increase in PM_2.5_ (lag 0–2 days) elevated mortality risks for esophageal, gastric, and colorectal cancers by 4%, 5%, and 4%, respectively (27), while a time-series study in Xi’an confirmed PM_2.5_’s association with gastric cancer mortality (RR = 1.0003) (28). Although our results align directionally with these studies, the effect magnitude is notably lower. Given the heterogeneity in genetic profiles, socioeconomic conditions, climatic factors, pollutant composition, and methodological approaches across existing investigations, current evidence remains insufficient to confirm whether adaptive mechanisms contribute to attenuated cancer mortality trends. Differences from the umbrella review (29) stem from exposure time scales and methods: we focused on short-term dynamic exposure (0–7 days) and acute mortality via time-stratified case-crossover (controlling confounders), whereas it used long-term static exposure (annual averages) and cohort studies for chronic incidence effects.

In terms of O_3_, this study provides novel evidence: Each 10 μg/m^3^ increase in O_3_ concentration at lag 0–5 days corresponds to a 0.84% elevation in GI cancer mortality risk. This finding echoes a Brazilian nationwide case-crossover study linking 8-h O_3_ exposure to increased all-cancer mortality, including gastric cancer (30). However, Chinese studies present conflicting results—while a study conducted in Guangzhou revealed positive O_3_-all-cancer mortality associations (31), the other two studies focusing on lung cancer showed null associations (32, 33). These inconsistencies may arise from: (1) methodological variations (case-crossover vs. time-series designs); (2) cancer-type specificity (all-cancer vs. single-site analyses); and (3) sample size limitations. Notably, this study pioneers verification of O_3_’s short-term health effects in a moderately polluted city (Yancheng’s daily mean O_3_: 104.89 μg/m^3^), offering new evidence for regional air quality standard revisions.

Regarding the lack of significant associations for SO_2_ and NO_2_, we attribute this primarily to Yancheng’s unique coastal atmospheric conditions: (1) prevailing southeastern winds enhance pollutant dispersion, yielding daily mean concentrations of 12.61 μg/m^3^ (SO_2_) and 23.89 μg/m^3^ (NO_2_)—significantly lower than industrial clusters like the Beijing-Tianjin-Hebei region; (2) marine-derived secondary aerosols may alter pollutant chemical profiles, potentially mitigating carcinogenicity (34). This low-concentration, low-variability exposure profile likely reduced statistical power, necessitating future multicenter studies to validate these findings’ generalizability.

Although the potential mechanisms linking ambient air pollutants to GI cancer mortality remain unclear, current research proposes a multi-pathway hypothesis. Particulate matter harbors carcinogenic constituents such as heavy metals and persistent organic pollutants, with demonstrated potential to initiate oncogenic processes and promote tumorigenesis through chronic exposure pathways. First, pollutants such as PM_2.5_ can enter the GI tract through dual routes: On one hand, inhaled PM_2.5_ partially crosses alveolar membranes into the bloodstream and deposits in intestinal tissues via systemic circulation (35); on the other hand, particles retained in bronchioles and alveoli are phagocytosed by macrophages (36) and subsequently transported to the upper GI tract through the mucociliary clearance mechanism (37, 38), a process confirmed in human studies of nonsmokers (39). Second, pollutants may directly disrupt intestinal barrier function: PM_2.5_ synergizes with toxic gasses like SO₂ and NOx to induce tight junction protein rearrangement in gut epithelial cells, increasing intestinal permeability (40), while its heavy metals and carcinogens may trigger localized oxidative stress and DNA damage. Third, the synergistic effects of systemic inflammation and microbial dysbiosis: PM exposure induces systemic inflammatory responses (40), and animal studies show air pollution alters gut microbiota composition, exacerbating susceptibility to mucosal inflammation (36, 41). This dual impact is particularly critical in GI cancer patients, who already exhibit chronic inflammation and microbial imbalance, potentially accelerating cancer progression. Notably, upper GI cancers (e.g., esophageal and gastric cancers) may have unique exposure patterns due to direct contact with PM cleared via mucus, though experimental evidence validating this hypothesis remains lacking.

Emerging evidence suggests that short-term O₃ exposure may elevate all-cancer mortality risk through interrelated pathways involving hemostatic imbalance, neuro-inflammatory activation, and systemic dysregulation. Short-term O₃ exposure induces a hypercoagulable state by upregulating coagulation factor X while suppressing anticoagulant proteins Z and ZPI, thereby increasing thrombotic susceptibility (42). Additionally, inhaled O₃ triggers sensory nerve stimulation in the respiratory tract, initiating local reflex reactions and propagating signals to the central nervous system. This neural activation disrupts autonomic function regulation, potentially exacerbating cardiovascular stress and systemic inflammation, which can lead to potential mortality (43). Concurrently, O₃ exposure promotes respiratory and systemic inflammation, which synergizes with coagulation abnormalities to amplify tissue damage and organ dysfunction and ultimately contributes to mortality in cancer patients. Clinically significant immune compromise in oncology populations, stemming from underlying malignancy and iatrogenic factors, amplifies susceptibility to airborne toxicants’ pathobiological effects, thereby elevating mortality risks. Moreover, emerging evidence identifies dysregulation of oncogenesis-associated mRNA/miRNA signatures following brief (≤2 h) low-concentration ambient pollutant exposures, suggesting accelerated carcinogenic pathways (44).

Current evidence on the modifying factors that influence the association between short-term exposure to air pollution and GI cancers is limited. Therefore, we conducted subgroup analyses to explore the potential modifying effects of sex, age, and tumor type. Our analyses revealed that the relationship between PM_2.5_, PM_10_, and O_3_ and GI cancer mortality is more robust in the older population, males, and patients with esophageal cancer. The heightened vulnerability of older adults aligns with age-related declines in immune resilience and increased comorbidity burden, which may exacerbate pollutant-induced oxidative damage. Most studies have found that women were more sensitive to the acute effects of air pollution, but our results contrast with this consensus, revealing stronger associations in males. This discrepancy may arise from sex-specific exposure patterns: in China, males exhibit a substantially higher smoking prevalence (47.2% in males vs. 2.7% in females (45)), which likely synergizes with air pollution to amplify acute cardiopulmonary stress and cancer progression. Furthermore, the robust association observed in esophageal cancer—a malignancy with rapid progression and shorter survival—contrasts with weaker effects in gastric, hepatic, and colorectal cancers. This is consistent with large cohort studies in Europe (46, 47). For malignancies with prolonged survival periods, such as gastric, hepatic, and colorectal cancers, patients may survive for years post-diagnosis, with eventual mortality often attributed to competing causes (e.g., cardiovascular events or infectious complications). When mortality alone is utilized as an endpoint, this approach may fail to capture cases where cancer contributes indirectly to death, thereby leading to a potential underestimation of the association between air pollutant exposure and carcinogenesis (23, 48).

This study pioneers the investigation of short-term air pollution effects on GI cancer mortality in a coastal area characterized by moderate pollution. Utilizing a robust methodology (case-crossover design combined with lagged modeling), it effectively controlled for individual confounders while capturing acute exposure risks. Furthermore, stratified analyses revealed higher susceptibility in the the older males and identified site-specific responses—for example, esophageal cancer was strongly linked to particulate matter, whereas colorectal cancer exhibited sensitivity to ozone. Despite its innovation, exposure misclassification from fixed-site pollution data may lead to an underestimation of individual risks. Additionally, unmeasured confounders (e.g., smoking, diet) and unclear biological mechanisms limit causal interpretation. Moreover, the findings may lack generalizability to high-pollution regions or other cancer types, emphasizing the need for broader validation.

## 5 Conclusion

This study demonstrates that short-term exposure to PM_2.5_, PM_10_, and O_3_ significantly increases the mortality risk of GI cancer patients in a moderately polluted coastal area. We also identified a higher susceptibility in males and the vulnerability of the older population, consistent with sex-specific inflammatory responses and age-related detoxification decline. The differences in anatomical timing—acute effects of PM on upper gastrointestinal cancers and delayed effects of O_3_ on lower gastrointestinal malignancies—reflect the observed patterns of pollutant deposition. These results necessitate urgent updates to air quality standards, emphasizing short-term exposure controls and targeted protections for susceptible populations to improve the survival of GI cancer patients.

## Data Availability

The original contributions presented in the study are included in the article/Supplementary material, further inquiries can be directed to the corresponding authors.

## Footnotes

^1^ http://data.cma.cn/

^2^ http://www.cnemc.cn/sssj/
