# Supplementary material for: Acute air pollution exposure and gastrointestinal cancer mortality: a case-crossover study in coastal China
Source: Front Public Health. 2025 Sep 25;13:1666928. doi: 10.3389/fpubh.2025.1666928 (PMC12507895; doi:10.3389/fpubh.2025.1666928)
Supplement: Supplementary file 1 [file Table_1.DOCX]

Table S1. Pearson’s correlation coefficients between air pollutants in Yancheng, China during 2013–2022.

|  | PM_2.5_ | PM_10_ | SO_2_ | NO_2_ | O_3_ | Temperature | Relative  humidity | Wind speed |
| --- | --- | --- | --- | --- | --- | --- | --- | --- |
| PM_2.5_ | 1 |  |  |  |  |  |  |  |
| PM_10_ | 0.913^***^ | 1 |  |  |  |  |  |  |
| SO_2_ | 0.567^***^ | 0.630^***^ | 1 |  |  |  |  |  |
| NO_2_ | 0.706^***^ | 0.731^***^ | 0.495^***^ | 1 |  |  |  |  |
| O_3_ | 0.030 ^NS^ | 0.061^***^ | 0.019^NS^ | -0.094^***^ | 1 |  |  |  |
| Temperature | -0.379^***^ | -0.375^***^ | -0.204^***^ | -0.460^***^ | 0.467^***^ | 1 |  |  |
| Relative humidity | -0.196^***^ | -0.368^***^ | -0.257^***^ | -0.269^***^ | -0.234^***^ | 0.221^***^ | 1 |  |
| Wind speed | -0.123^***^ | -0.086^***^ | 0.105^***^ | -0.311^***^ | -0.050^**^ | 0.022^NS^ | -0.050^**^ | 1 |

Note: *. Correlation is significant at the 0.05 level (2-tailed); **. Correlation is significant at the 0.01 level (2-tailed); ***. Correlation is significant at the 0.001 level (2-tailed); NS, no significant.

Table S2. The lag-specific RRs and 95% CIs of GI cancer deaths associated with a 10μg/m^3^ increase of PM_2.5_, PM_10_, SO_2_, NO_2_, and O_3_ in age-stratified analysis: aged <65 years.

| Lag days | PM_2.5_ (RR [95%CI]) |  | PM_10_ (RR [95%CI]) |  | SO_2_ (RR [95%CI]) |  | NO_2_ (RR [95%CI]) |  | O_3_ (RR [95%CI]) |
| --- | --- | --- | --- | --- | --- | --- | --- | --- | --- |
| lag 0 | 1.0019(0.9966,1.0072) |  | 1.0025(0.9986,1.0064) |  | 0.9956(0.9748,1.0169) |  | 1.0295(0.9386,1.1293) |  | 0.9987(0.9948,1.0026) |
| lag 1 | 1.0012(0.9968,1.0055) |  | 1.0019(0.9987,1.0051) |  | 0.9958(0.9790,1.0129) |  | 1.0274(0.9509,1.1099) |  | 0.9988(0.9957,1.0020) |
| lag 2 | 1.0005(0.9969,1.0040) |  | 1.0014(0.9988,1.0039) |  | 0.9960(0.9827,1.0094) |  | 1.0252(0.9604,1.0944) |  | 0.9990(0.9964,1.0016) |
| lag 3 | 0.9998(0.9967,1.0029) |  | 1.0008(0.9987,1.0030) |  | 0.9962(0.9853,1.0071) |  | 1.0231(0.9649,1.0847) |  | 0.9991(0.9969,1.0014) |
| lag 4 | 0.9991(0.9960,1.0021) |  | 1.0003(0.9981,1.0024) |  | 0.9963(0.9860,1.0068) |  | 1.0209(0.9627,1.0827) |  | 0.9993(0.9971,1.0015) |
| lag 5 | 0.9984(0.9948,1.0019) |  | 0.9997(0.9972,1.0022) |  | 0.9965(0.9845,1.0087) |  | 1.0188(0.9539,1.0881) |  | 0.9994(0.9969,1.0020) |
| lag 6 | 0.9977(0.9934,1.0019) |  | 0.9991(0.9961,1.0022) |  | 0.9967(0.9816,1.0121) |  | 1.0166(0.9404,1.0991) |  | 0.9996(0.9964,1.0028) |
| lag 7 | 0.9970(0.9918,1.0022) |  | 0.9986(0.9948,1.0024) |  | 0.9969(0.9779,1.0162) |  | 1.0145(0.9242,1.1137) |  | 0.9997(0.9958,1.0037) |
| lag 01 | 1.0030(0.9934,1.0127) |  | 1.0044(0.9973,1.0116) |  | 0.9914(0.9544,1.0298) |  | 1.0577(0.8931,1.2527) |  | 0.9975(0.9905,1.0047) |
| lag 02 | 1.0035(0.9905,1.0167) |  | 1.0058(0.9963,1.0155) |  | 0.9874(0.9384,1.0390) |  | 1.0844(0.8599,1.3674) |  | 0.9965(0.9870,1.0062) |
| lag 03 | 1.0033(0.9876,1.0191) |  | 1.0066(0.9952,1.0182) |  | 0.9836(0.9260,1.0449) |  | 1.1094(0.8357,1.4726) |  | 0.9957(0.9843,1.0072) |
| lag 04 | 1.0023(0.9846,1.0204) |  | 1.0069(0.9941,1.0198) |  | 0.9800(0.9165,1.0479) |  | 1.1326(0.8169,1.5702) |  | 0.9950(0.9822,1.0079) |
| lag 05 | 1.0007(0.9811,1.0206) |  | 1.0066(0.9927,1.0207) |  | 0.9766(0.9089,1.0494) |  | 1.1538(0.7995,1.6653) |  | 0.9944(0.9805,1.0086) |
| lag 06 | 0.9983(0.9769,1.0202) |  | 1.0057(0.9906,1.0210) |  | 0.9734(0.9016,1.0508) |  | 1.1730(0.7787,1.7671) |  | 0.9940(0.9788,1.0095) |
| lag 07 | 0.9953(0.9715,1.0197) |  | 1.0043(0.9877,1.0212) |  | 0.9703(0.8929,1.0545) |  | 1.1901(0.7501,1.8881) |  | 0.9938(0.9768,1.0110) |

Note: The bold number indicates the *P*<0.05.

RR, relative ratio; CI, confidence interval.

NO_2_, nitrogen dioxide; O_3,_ ozone; PM_2.5_, particulate matter with an aerodynamic diameter <2.5 mm; PM_10_, particulate matter with an aerodynamic diameter <10 mm; SO_2_, sulfur dioxide.

Table S3. The lag-specific RRs and 95% CIs of GI cancer deaths associated with a 10μg/m^3^ increase of PM_2.5_, PM_10_, SO_2_, NO_2_, and O_3_ in age-stratified analysis: aged ≥65 years.

| Lag days | PM_2.5_ (RR [95%CI]) |  | PM_10_ (RR [95%CI]) |  | SO_2_ (RR [95%CI]) |  | NO_2_ (RR [95%CI]) |  | O_3_ (RR [95%CI]) |
| --- | --- | --- | --- | --- | --- | --- | --- | --- | --- |
| lag 0 | **1.0037(1.0002,1.0073)** |  | **1.0030(1.0004,1.0056)** |  | 1.0060(0.9906,1.0217) |  | 0.9728(0.9153,1.0339) |  | **1.0038(1.0013,1.0064)** |
| lag 1 | **1.0033(1.0004,1.0062)** |  | **1.0025(1.0004,1.0046)** |  | 1.0060(0.9936,1.0185) |  | 0.9834(0.9345,1.0349) |  | **1.0032(1.0011,1.0053)** |
| lag 2 | **1.0028(1.0004,1.0052)** |  | **1.0019(1.0002,1.0037)** |  | 1.0059(0.9962,1.0158) |  | 0.9941(0.9521,1.0379) |  | **1.0026(1.0009,1.0043)** |
| lag 3 | **1.0024(1.0003,1.0045)** |  | 1.0014(1.0000,1.0029) |  | 1.0059(0.9979,1.0139) |  | 1.0049(0.9669,1.0445) |  | **1.0020(1.0006,1.0035)** |
| lag 4 | 1.0020(0.9999,1.0040) |  | 1.0009(0.9995,1.0023) |  | 1.0058(0.9981,1.0136) |  | 1.0159(0.9774,1.0558) |  | 1.0014(1.0000,1.0029) |
| lag 5 | 1.0015(0.9991,1.0039) |  | 1.0004(0.9987,1.0020) |  | 1.0058(0.9967,1.0150) |  | 1.0269(0.9836,1.0721) |  | 1.0008(0.9991,1.0025) |
| lag 6 | 1.0011(0.9982,1.0039) |  | 0.9998(0.9978,1.0019) |  | 1.0057(0.9942,1.0174) |  | 1.0381(0.9866,1.0923) |  | 1.0002(0.9981,1.0023) |
| lag 7 | 1.0006(0.9971,1.0041) |  | 0.9993(0.9968,1.0019) |  | 1.0057(0.9912,1.0204) |  | 1.0494(0.9875,1.1152) |  | 0.9996(0.9971,1.0022) |
| lag 01 | **1.0070(1.0005,1.0135)** |  | **1.0055(1.0008,1.0103)** |  | 1.0120(0.9843,1.0405) |  | 0.9567(0.8557,1.0696) |  | **1.0071(1.0024,1.0118)** |
| lag 02 | **1.0099(1.0011,1.0188)** |  | **1.0075(1.0011,1.0139)** |  | 1.0180(0.9809,1.0565) |  | 0.9510(0.8161,1.1083) |  | **1.0098(1.0034,1.0161)** |
| lag 03 | **1.0123(1.0017,1.0230)** |  | **1.0089(1.0013,1.0166)** |  | 1.0240(0.9800,1.0699) |  | 0.9557(0.7927,1.1523) |  | **1.0118(1.0042,1.0194)** |
| lag 04 | **1.0143(1.0022,1.0265)** |  | **1.0098(1.0012,1.0184)** |  | 1.0299(0.9810,1.0813) |  | 0.9709(0.7825,1.2046) |  | **1.0132(1.0047,1.0219)** |
| lag 05 | **1.0158(1.0024,1.0294)** |  | **1.0101(1.0008,1.0195)** |  | 1.0359(0.9832,1.0914) |  | 0.9970(0.7826,1.2703) |  | **1.0141(1.0047,1.0235)** |
| lag 06 | **1.0169(1.0022,1.0318)** |  | 1.0100(0.9999,1.0202) |  | 1.0418(0.9852,1.1017) |  | 1.0350(0.7899,1.3561) |  | **1.0143(1.0040,1.0247)** |
| lag 07 | **1.0175(1.0011,1.0342)** |  | 1.0093(0.9981,1.0206) |  | 1.0478(0.9857,1.1138) |  | 1.0861(0.8015,1.4718) |  | **1.0139(1.0024,1.0255)** |

Note: The bold number indicates the *P*<0.05.

RR, relative ratio; CI, confidence interval.

NO_2_, nitrogen dioxide; O_3,_ ozone;PM_2.5_, particulate matter with an aerodynamic diameter <2.5 mm; PM_10_, particulate matter with an aerodynamic diameter <10 mm; SO_2_, sulfur dioxide.

Table S4. The lag-specific RRs and 95% CIs of GI cancer deaths associated with a 10μg/m^3^ increase of PM_2.5,_ PM_10_, SO_2_, NO_2_, and O_3_ in sex-stratified analysis: male.

| Lag days | PM_2.5_ (RR [95%CI]) |  | PM_10_ (RR [95%CI]) |  | SO_2_ (RR [95%CI]) |  | NO_2_ (RR [95%CI]) |  | O_3_ (RR [95%CI]) |
| --- | --- | --- | --- | --- | --- | --- | --- | --- | --- |
| lag 0 | **1.0046(1.0009,1.0082)** |  | **1.0041(1.0014,1.0068)** |  | 1.0011(0.9861,1.0164) |  | 0.9777(0.9182,1.0410) |  | **1.0027(1.0001,1.0054)** |
| lag 1 | **1.0039(1.0009,1.0069)** |  | **1.0032(1.0010,1.0054)** |  | 1.0015(0.9895,1.0137) |  | 0.9844(0.9340,1.0375) |  | **1.0023(1.0002,1.0045)** |
| lag 2 | **1.0033(1.0008,1.0057)** |  | **1.0023(1.0006,1.0041)** |  | 1.0019(0.9924,1.0115) |  | 0.9911(0.9481,1.0361) |  | **1.0019(1.0002,1.0036)** |
| lag 3 | **1.0027(1.0005,1.0048)** |  | 1.0014(1.0000,1.0029) |  | 1.0023(0.9945,1.0102) |  | 0.9979(0.9591,1.0383) |  | 1.0015(1.0000,1.0030) |
| lag 4 | 1.0020(0.9999,1.0041) |  | 1.0006(0.9991,1.0020) |  | 1.0027(0.9951,1.0103) |  | 1.0048(0.9657,1.0454) |  | 1.0010(0.9996,1.0025) |
| lag 5 | 1.0014(0.9990,1.0038) |  | 0.9997(0.9980,1.0014) |  | 1.0031(0.9941,1.0121) |  | 1.0117(0.9678,1.0575) |  | 1.0006(0.9989,1.0024) |
| lag 6 | 1.0008(0.9978,1.0037) |  | 0.9988(0.9967,1.0009) |  | 1.0035(0.9921,1.0150) |  | 1.0186(0.9666,1.0734) |  | 1.0002(0.9981,1.0023) |
| lag 7 | 1.0001(0.9966,1.0037) |  | 0.9980(0.9954,1.0006) |  | 1.0039(0.9896,1.0183) |  | 1.0256(0.9633,1.0918) |  | 0.9998(0.9971,1.0024) |
| lag 01 | **1.0085(1.0019,1.0151)** |  | **1.0073(1.0024,1.0121)** |  | 1.0015(0.9895,1.0137) |  | 0.9624(0.8579,1.0796) |  | **1.0051(1.0003,1.0099)** |
| lag 02 | **1.0118(1.0028,1.0209)** |  | **1.0096(1.0031,1.0162)** |  | 1.0019(0.9924,1.0115) |  | 0.9539(0.8148,1.1167) |  | **1.0070(1.0005,1.0135)** |
| lag 03 | **1.0145(1.0036,1.0255)** |  | **1.0111(1.0033,1.0189)** |  | 1.0023(0.9945,1.0102) |  | 0.9519(0.7852,1.1539) |  | **1.0085(1.0007,1.0163)** |
| lag 04 | **1.0165(1.0042,1.0290)** |  | **1.0116(1.0029,1.0205)** |  | 1.0027(0.9951,1.0103) |  | 0.9564(0.7661,1.1941) |  | **1.0095(1.0008,1.0184)** |
| lag 05 | **1.0179(1.0043,1.0318)** |  | **1.0113(1.0018,1.0209)** |  | 1.0031(0.9941,1.0121) |  | 0.9676(0.7543,1.2412) |  | **1.0102(1.0005,1.0199)** |
| lag 06 | **1.0187(1.0037,1.0340)** |  | 1.0102(0.9998,1.0206) |  | 1.0035(0.9921,1.0150) |  | 0.9856(0.7466,1.3010) |  | 1.0103(0.9998,1.0210) |
| lag 07 | **1.0188(1.0021,1.0359)** |  | 1.0081(0.9967,1.0196) |  | 1.0039(0.9896,1.0183) |  | 1.0108(0.7397,1.3812) |  | 1.0101(0.9984,1.0220) |

Note: The bold number indicates the *P*<0.05.

RR, relative ratio; CI, confidence interval.

NO_2_, nitrogen dioxide; O_3,_ ozone; PM_2.5_, particulate matter with an aerodynamic diameter <2.5 mm; PM_10_, particulate matter with an aerodynamic diameter <10 mm; SO_2_, sulfur dioxide.

Table S5. The lag-specific RRs and 95% CIs of GI cancer deaths associated with a 10μg/m^3^ increase of PM_2.5_, PM_10_, SO_2_, NO_2_, and O_3_ in sex-stratified analysis: female.

| Lag days | PM_2.5_ (RR [95%CI]) |  | PM_10_ (RR [95%CI]) |  | SO_2_ (RR [95%CI]) |  | NO_2_ (RR [95%CI]) |  | O_3_ (RR [95%CI]) |
| --- | --- | --- | --- | --- | --- | --- | --- | --- | --- |
| lag 0 | 1.0004(0.9954,1.0054) |  | 1.0006(0.9969,1.0042) |  | 1.0056(0.9841,1.0274) |  | 1.0067(0.9249,1.0957) |  | 1.0017(0.9981,1.0053) |
| lag 1 | 1.0001(0.9961,1.0042) |  | 1.0007(0.9977,1.0037) |  | 1.0048(0.9876,1.0224) |  | 1.0156(0.9460,1.0902) |  | 1.0013(0.9984,1.0043) |
| lag 2 | 0.9999(0.9966,1.0033) |  | 1.0008(0.9984,1.0032) |  | 1.0041(0.9905,1.0179) |  | 1.0245(0.9648,1.0880) |  | 1.0010(0.9986,1.0034) |
| lag 3 | 0.9997(0.9968,1.0026) |  | 1.0009(0.9989,1.0029) |  | 1.0034(0.9923,1.0146) |  | 1.0336(0.9793,1.0909) |  | 1.0006(0.9986,1.0027) |
| lag 4 | 0.9994(0.9966,1.0023) |  | 1.0010(0.9991,1.0030) |  | 1.0026(0.9921,1.0132) |  | 1.0427(0.9878,1.1007) |  | 1.0003(0.9983,1.0023) |
| lag 5 | 0.9992(0.9959,1.0025) |  | 1.0012(0.9989,1.0035) |  | 1.0019(0.9897,1.0142) |  | 1.0520(0.9902,1.1176) |  | 0.9999(0.9976,1.0023) |
| lag 6 | 0.9989(0.9950,1.0029) |  | 1.0013(0.9984,1.0041) |  | 1.0012(0.9859,1.0167) |  | 1.0612(0.9879,1.1400) |  | 0.9996(0.9967,1.0025) |
| lag 7 | 0.9987(0.9938,1.0036) |  | 1.0014(0.9978,1.0050) |  | 1.0004(0.9812,1.0201) |  | 1.0706(0.9829,1.1661) |  | 0.9992(0.9956,1.0028) |
| lag 01 | 1.0005(0.9916,1.0096) |  | 1.0013(0.9947,1.0079) |  | 1.0104(0.9720,1.0503) |  | 1.0224(0.8755,1.1939) |  | 1.0030(0.9965,1.0096) |
| lag 02 | 1.0004(0.9883,1.0127) |  | 1.0021(0.9932,1.0110) |  | 1.0145(0.9632,1.0686) |  | 1.0475(0.8467,1.2959) |  | 1.0040(0.9953,1.0129) |
| lag 03 | 1.0001(0.9855,1.0149) |  | 1.0030(0.9924,1.0137) |  | 1.0180(0.9572,1.0826) |  | 1.0826(0.8346,1.4044) |  | 1.0047(0.9942,1.0153) |
| lag 04 | 0.9995(0.9830,1.0164) |  | 1.0040(0.9922,1.0160) |  | 1.0206(0.9533,1.0927) |  | 1.1289(0.8360,1.5245) |  | 1.0050(0.9931,1.0170) |
| lag 05 | 0.9987(0.9805,1.0173) |  | 1.0052(0.9923,1.0183) |  | 1.0226(0.9505,1.1001) |  | 1.1876(0.8472,1.6647) |  | 1.0049(0.9919,1.0181) |
| lag 06 | 0.9977(0.9776,1.0181) |  | 1.0065(0.9925,1.0207) |  | 1.0238(0.9472,1.1065) |  | 1.2603(0.8641,1.8382) |  | 1.0045(0.9903,1.0189) |
| lag 07 | 0.9964(0.9740,1.0192) |  | 1.0079(0.9924,1.0236) |  | 1.0242(0.9415,1.1142) |  | 1.3493(0.8819,2.0645) |  | 1.0037(0.9879,1.0198) |

Note: The bold number indicates the *P*<0.05.

RR, relative ratio; CI, confidence interval.

NO_2_, nitrogen dioxide; O_3,_ ozone; PM_2.5_, particulate matter with an aerodynamic diameter <2.5 mm; PM_10_, particulate matter with an aerodynamic diameter <10 mm; SO_2_, sulfur dioxide.

Table S6. The lag-specific RRs and 95% CIs of GI cancer deaths associated with a 10μg/m^3^ increase of PM_2.5_, PM_10_, SO_2_, NO_2_, and O_3_ in cancer-stratified analysis: Esophagus cancer.

| Lag days | PM_2.5_ (RR [95%CI]) |  | PM_10_ (RR [95%CI]) |  | SO_2_ (RR [95%CI]) |  | NO_2_ (RR [95%CI]) |  | O_3_ (RR [95%CI]) |
| --- | --- | --- | --- | --- | --- | --- | --- | --- | --- |
| lag 0 | **1.0076(1.0022,1.0130)** |  | **1.0073(1.0034,1.0113)** |  | 0.9861(0.9644,1.0084) |  | 0.9016(0.8213,0.9898) |  | 1.0039(1.0000,1.0078) |
| lag 1 | **1.0059(1.0015,1.0103)** |  | **1.0057(1.0025,1.0089)** |  | 0.9917(0.9742,1.0096) |  | 0.9280(0.8583,1.0035) |  | **1.0032(1.0000,1.0063)** |
| lag 2 | **1.0043(1.0007,1.0079)** |  | **1.0041(1.0016,1.0067)** |  | 0.9974(0.9835,1.0114) |  | 0.9552(0.8942,1.0204) |  | 1.0025(0.9999,1.0050) |
| lag 3 | 1.0027(0.9995,1.0058) |  | **1.0025(1.0004,1.0047)** |  | 1.0030(0.9916,1.0146) |  | 0.9832(0.9273,1.0424) |  | 1.0018(0.9996,1.0040) |
| lag 4 | 1.0010(0.9979,1.0041) |  | 1.0010(0.9988,1.0031) |  | 1.0087(0.9977,1.0199) |  | 1.0120(0.9553,1.0721) |  | 1.0011(0.9989,1.0033) |
| lag 5 | 0.9994(0.9959,1.0029) |  | 0.9994(0.9969,1.0018) |  | 1.0144(1.0014,1.0277) |  | 1.0416(0.9773,1.1102) |  | 1.0004(0.9978,1.0029) |
| lag 6 | 0.9977(0.9935,1.0020) |  | 0.9978(0.9947,1.0009) |  | 1.0202(1.0035,1.0372) |  | 1.0722(0.9947,1.1557) |  | 0.9997(0.9965,1.0028) |
| lag 7 | 0.9961(0.9909,1.0013) |  | 0.9962(0.9924,1.0000) |  | 1.0260(1.0049,1.0476) |  | **1.1036(1.0090,1.2070)** |  | 0.9990(0.9951,1.0028) |
| lag 01 | **1.0136(1.0038,1.0234)** |  | **1.0131(1.0059,1.0203)** |  | 0.9780(0.9396,1.0179) |  | **0.8367(0.7053,0.9927)** |  | **1.0071(1.0000,1.0142)** |
| lag 02 | **1.0179(1.0046,1.0313)** |  | **1.0173(1.0076,1.0270)** |  | 0.9754(0.9246,1.0290) |  | 0.7993(0.6322,1.0105) |  | **1.0095(1.0000,1.0192)** |
| lag 03 | **1.0206(1.0046,1.0369)** |  | **1.0198(1.0083,1.0316)** |  | 0.9784(0.9184,1.0423) |  | 0.7858(0.5901,1.0465) |  | 1.0113(0.9999,1.0229) |
| lag 04 | **1.0216(1.0035,1.0401)** |  | **1.0208(1.0078,1.0340)** |  | 0.9869(0.9202,1.0585) |  | 0.7953(0.5718,1.1060) |  | 1.0124(0.9995,1.0254) |
| lag 05 | **1.0210(1.0010,1.0414)** |  | **1.0202(1.0061,1.0345)** |  | 1.0012(0.9288,1.0792) |  | 0.8284(0.5728,1.1981) |  | 1.0127(0.9986,1.0271) |
| lag 06 | 1.0187(0.9968,1.0412) |  | **1.0179(1.0026,1.0334)** |  | 1.0214(0.9429,1.1065) |  | 0.8882(0.5897,1.3377) |  | 1.0124(0.9969,1.0281) |
| lag 07 | 1.0148(0.9904,1.0397) |  | 1.0140(0.9972,1.0311) |  | 1.0480(0.9604,1.1435) |  | 0.9801(0.6203,1.5487) |  | 1.0113(0.9941,1.0289) |

Note: The bold number indicates the *P*<0.05.

RR, relative ratio; CI, confidence interval.

NO_2_, nitrogen dioxide; O_3,_ ozone; PM_2.5_, particulate matter with an aerodynamic diameter <2.5 mm; PM_10_, particulate matter with an aerodynamic diameter <10 mm; SO_2_, sulfur dioxide.

Table S7. The lag-specific RRs and 95% CIs of GI cancer deaths associated with a 10μg/m^3^ increase of PM_2.5_, PM_10_, SO_2_, NO_2_, and O_3_ in cancer-stratified analysis: Stomach cancer.

| Lag days | PM_2.5_ (RR [95%CI]) |  | PM_10_ (RR [95%CI]) |  | SO_2_ (RR [95%CI]) |  | NO_2_ (RR [95%CI]) |  | O_3_ (RR [95%CI]) |
| --- | --- | --- | --- | --- | --- | --- | --- | --- | --- |
| lag 0 | 1.0026(0.9971,1.0081) |  | 1.0018(0.9978,1.0059) |  | 1.0077(0.9856,1.0303) |  | 1.0046(0.9141,1.1039) |  | 0.9989(0.9949,1.0030) |
| lag 1 | 1.0024(0.9979,1.0070) |  | 1.0016(0.9983,1.0049) |  | 1.0063(0.9886,1.0244) |  | 1.0081(0.9317,1.0908) |  | 0.9992(0.9959,1.0025) |
| lag 2 | 1.0023(0.9986,1.0060) |  | 1.0013(0.9987,1.0040) |  | 1.0049(0.9910,1.0191) |  | 1.0117(0.9465,1.0814) |  | 0.9995(0.9969,1.0022) |
| lag 3 | 1.0021(0.9989,1.0054) |  | 1.0011(0.9989,1.0033) |  | 1.0036(0.9920,1.0152) |  | 1.0153(0.9563,1.0779) |  | 0.9998(0.9975,1.0021) |
| lag 4 | 1.0020(0.9988,1.0052) |  | 1.0008(0.9986,1.0030) |  | 1.0022(0.9909,1.0136) |  | 1.0189(0.9592,1.0823) |  | 1.0001(0.9978,1.0024) |
| lag 5 | 1.0018(0.9982,1.0055) |  | 1.0006(0.9980,1.0032) |  | 1.0008(0.9875,1.0142) |  | 1.0225(0.9553,1.0945) |  | 1.0004(0.9978,1.0031) |
| lag 6 | 1.0017(0.9972,1.0062) |  | 1.0003(0.9971,1.0035) |  | 0.9994(0.9827,1.0164) |  | 1.0262(0.9466,1.1124) |  | 1.0007(0.9974,1.0040) |
| lag 7 | 1.0016(0.9961,1.0070) |  | 1.0001(0.9961,1.0041) |  | 0.9980(0.9771,1.0194) |  | 1.0298(0.9350,1.1342) |  | 1.0010(0.9970,1.0050) |
| lag 01 | 1.0050(0.9950,1.0152) |  | 1.0034(0.9961,1.0108) |  | 1.0141(0.9745,1.0553) |  | 1.0127(0.8523,1.2034) |  | 0.9982(0.9909,1.0055) |
| lag 02 | 1.0073(0.9937,1.0211) |  | 1.0048(0.9949,1.0147) |  | 1.0191(0.9663,1.0749) |  | 1.0246(0.8089,1.2978) |  | 0.9977(0.9879,1.0076) |
| lag 03 | 1.0095(0.9930,1.0262) |  | 1.0059(0.9941,1.0178) |  | 1.0227(0.9602,1.0894) |  | 1.0402(0.7794,1.3883) |  | 0.9975(0.9858,1.0094) |
| lag 04 | 1.0115(0.9928,1.0305) |  | 1.0067(0.9935,1.0201) |  | 1.0250(0.9556,1.0994) |  | 1.0599(0.7597,1.4787) |  | 0.9976(0.9844,1.0110) |
| lag 05 | 1.0134(0.9927,1.0345) |  | 1.0073(0.9929,1.0219) |  | 1.0258(0.9513,1.1062) |  | 1.0838(0.7453,1.5759) |  | 0.9980(0.9836,1.0127) |
| lag 06 | 1.0151(0.9923,1.0384) |  | 1.0076(0.9920,1.0235) |  | 1.0252(0.9454,1.1117) |  | 1.1121(0.7316,1.6907) |  | 0.9987(0.9829,1.0148) |
| lag 07 | 1.0167(0.9912,1.0428) |  | 1.0077(0.9904,1.0253) |  | 1.0232(0.9361,1.1185) |  | 1.1453(0.7136,1.8380) |  | 0.9997(0.9821,1.0177) |

Note: The bold number indicates the *P*<0.05.

RR, relative ratio; CI, confidence interval.

NO_2_, nitrogen dioxide; O_3_, ozone; PM_2.5_, particulate matter with an aerodynamic diameter <2.5 mm; PM_10_, particulate matter with an aerodynamic diameter <10 mm; SO_2_, sulfur dioxide.

Table S8. The lag-specific RRs and 95% CIs of GI cancer deaths associated with a 10μg/m^3^ increase of PM_2.5_, PM_10_, SO_2_, NO_2_, and O_3_ in cancer-stratified analysis: Liver cancer.

| Lag days | PM_2.5_ (RR [95%CI]) |  | PM_10_ (RR [95%CI]) |  | SO_2_ (RR [95%CI]) |  | NO_2_ (RR [95%CI]) |  | O_3_ (RR [95%CI]) |
| --- | --- | --- | --- | --- | --- | --- | --- | --- | --- |
| lag 0 | 1.0007(0.9944,1.0070) |  | 0.9993(0.9946,1.0039) |  | 1.0121(0.9858,1.0392) |  | 1.0464(0.9361,1.1698) |  | 1.0009(0.9963,1.0056) |
| lag 1 | 1.0009(0.9957,1.0060) |  | 0.9995(0.9957,1.0033) |  | 1.0074(0.9862,1.0291) |  | 1.0326(0.9412,1.1329) |  | 1.0007(0.9969,1.0044) |
| lag 2 | 1.0010(0.9967,1.0053) |  | 0.9997(0.9967,1.0027) |  | 1.0027(0.9859,1.0197) |  | 1.0189(0.9426,1.1014) |  | 1.0004(0.9973,1.0034) |
| lag 3 | 1.0011(0.9974,1.0048) |  | 0.9999(0.9973,1.0024) |  | 0.9979(0.9842,1.0119) |  | 1.0054(0.9379,1.0778) |  | 1.0001(0.9975,1.0027) |
| lag 4 | 1.0013(0.9976,1.0050) |  | 1.0001(0.9976,1.0026) |  | 0.9932(0.9802,1.0064) |  | 0.9921(0.9250,1.0641) |  | 0.9998(0.9972,1.0024) |
| lag 5 | 1.0014(0.9972,1.0056) |  | 1.0003(0.9974,1.0032) |  | 0.9886(0.9737,1.0037) |  | 0.9790(0.9043,1.0597) |  | 0.9995(0.9964,1.0025) |
| lag 6 | 1.0015(0.9965,1.0066) |  | 1.0005(0.9969,1.0041) |  | 0.9839(0.9654,1.0028) |  | 0.9660(0.8787,1.0619) |  | 0.9992(0.9954,1.0030) |
| lag 7 | 1.0017(0.9955,1.0079) |  | 1.0007(0.9962,1.0052) |  | 0.9793(0.9562,1.0029) |  | 0.9532(0.8507,1.0680) |  | 0.9989(0.9943,1.0036) |
| lag 01 | 1.0016(0.9902,1.0131) |  | 0.9988(0.9904,1.0072) |  | 1.0196(0.9723,1.0693) |  | 1.0806(0.8817,1.3243) |  | 1.0016(0.9932,1.0101) |
| lag 02 | 1.0026(0.9871,1.0182) |  | 0.9985(0.9872,1.0098) |  | 1.0223(0.9591,1.0897) |  | 1.1010(0.8337,1.4540) |  | 1.0020(0.9907,1.0134) |
| lag 03 | 1.0037(0.9851,1.0227) |  | 0.9983(0.9850,1.0119) |  | 1.0202(0.9457,1.1006) |  | 1.1069(0.7890,1.5530) |  | 1.0020(0.9885,1.0157) |
| lag 04 | 1.0050(0.9838,1.0266) |  | 0.9984(0.9834,1.0136) |  | 1.0133(0.9312,1.1026) |  | 1.0982(0.7440,1.6210) |  | 1.0018(0.9866,1.0172) |
| lag 05 | 1.0064(0.9830,1.0303) |  | 0.9987(0.9824,1.0153) |  | 1.0017(0.9147,1.0971) |  | 1.0751(0.6949,1.6632) |  | 1.0013(0.9846,1.0182) |
| lag 06 | 1.0080(0.9822,1.0344) |  | 0.9992(0.9815,1.0172) |  | 0.9856(0.8944,1.0862) |  | 1.0385(0.6382,1.6899) |  | 1.0005(0.9823,1.0190) |
| lag 07 | 1.0097(0.9809,1.0393) |  | 0.9999(0.9803,1.0199) |  | 0.9652(0.8686,1.0727) |  | 0.9899(0.5716,1.7142) |  | 0.9994(0.9791,1.0201) |

Note: The bold number indicates the *P*<0.05.

RR, relative ratio; CI, confidence interval.

NO_2_, nitrogen dioxide; O_3_, ozone; PM_2.5_, particulate matter with an aerodynamic diameter <2.5 mm; PM_10_, particulate matter with an aerodynamic diameter <10 mm; SO_2_, sulfur dioxide.

Table S9. The lag-specific RRs and 95% CIs of GI cancer deaths associated with a 10μg/m^3^ increase of PM_2.5_, PM_10_, SO_2_, NO_2_, and O_3_ in cancer-stratified analysis: Colorectum cancer.

| Lag days | PM_2.5_ (RR [95%CI]) |  | PM_10_ (RR [95%CI]) |  | SO_2_ (RR [95%CI]) |  | NO_2_ (RR [95%CI]) |  | O_3_ (RR [95%CI]) |
| --- | --- | --- | --- | --- | --- | --- | --- | --- | --- |
| lag 0 | 0.9985(0.9895,1.0077) |  | 0.9989(0.9922,1.0057) |  | 1.0224(0.9774,1.0696) |  | 1.0799(0.9241,1.2620) |  | 1.0060(0.9995,1.0126) |
| lag 1 | 0.9986(0.9912,1.0061) |  | 0.9993(0.9939,1.0048) |  | 1.0167(0.9811,1.0536) |  | 1.0752(0.9439,1.2248) |  | **1.0054(1.0001,1.0108)** |
| lag 2 | 0.9987(0.9926,1.0049) |  | 0.9997(0.9954,1.0041) |  | 1.0110(0.9835,1.0394) |  | 1.0706(0.9591,1.1950) |  | **1.0048(1.0005,1.0091)** |
| lag 3 | 0.9988(0.9935,1.0042) |  | 1.0001(0.9964,1.0038) |  | 1.0054(0.9831,1.0282) |  | 1.0659(0.9663,1.1759) |  | **1.0042(1.0005,1.0079)** |
| lag 4 | 0.9989(0.9936,1.0042) |  | 1.0005(0.9969,1.0042) |  | 0.9998(0.9779,1.0221) |  | 1.0613(0.9622,1.1707) |  | 1.0035(0.9999,1.0072) |
| lag 5 | 0.9990(0.9930,1.0051) |  | 1.0009(0.9967,1.0052) |  | 0.9942(0.9678,1.0213) |  | 1.0567(0.9469,1.1793) |  | 1.0029(0.9987,1.0072) |
| lag 6 | 0.9991(0.9918,1.0065) |  | 1.0013(0.9960,1.0066) |  | 0.9886(0.9548,1.0236) |  | 1.0521(0.9239,1.1982) |  | 1.0023(0.9971,1.0076) |
| lag 7 | 0.9992(0.9902,1.0083) |  | 1.0017(0.9951,1.0083) |  | 0.9831(0.9407,1.0273) |  | 1.0476(0.8967,1.2238) |  | 1.0017(0.9953,1.0082) |
| lag 01 | 0.9972(0.9808,1.0138) |  | 0.9983(0.9862,1.0105) |  | 1.0396(0.9592,1.1267) |  | 1.1612(0.8731,1.5443) |  | 1.0114(0.9996,1.0235) |
| lag 02 | 0.9959(0.9739,1.0185) |  | 0.9980(0.9817,1.0145) |  | 1.0510(0.9444,1.1697) |  | 1.2431(0.8410,1.8375) |  | **1.0163(1.0002,1.0326)** |
| lag 03 | 0.9948(0.9683,1.0220) |  | 0.9981(0.9788,1.0178) |  | 1.0567(0.9320,1.1981) |  | 1.3251(0.8224,2.1351) |  | **1.0205(1.0012,1.0402)** |
| lag 04 | 0.9937(0.9637,1.0247) |  | 0.9986(0.9770,1.0208) |  | 1.0564(0.9202,1.2129) |  | 1.4063(0.8116,2.4367) |  | **1.0241(1.0023,1.0464)** |
| lag 05 | 0.9927(0.9597,1.0270) |  | 0.9996(0.9760,1.0237) |  | 1.0503(0.9066,1.2167) |  | 1.4861(0.8022,2.7528) |  | **1.0271(1.0031,1.0517)** |
| lag 06 | 0.9919(0.9556,1.0296) |  | 1.0009(0.9753,1.0271) |  | 1.0383(0.8877,1.2146) |  | 1.5636(0.7865,3.1085) |  | **1.0295(1.0032,1.0565)** |
| lag 07 | 0.9911(0.9506,1.0333) |  | 1.0026(0.9742,1.0318) |  | 1.0208(0.8592,1.2128) |  | 1.6380(0.7565,3.5465) |  | **1.0312(1.0019,1.0614)** |

Note: The bold number indicates the *P*<0.05.

RR, relative ratio; CI, confidence interval.

NO_2_, nitrogen dioxide; O_3_, ozone; PM_2.5_, particulate matter with an aerodynamic diameter <2.5 mm; PM_10_, particulate matter with an aerodynamic diameter <10 mm; SO_2_, sulfur dioxide.

Table S10. The lag-specific RRs and 95% CIs of GI cancer deaths associated with a 10μg/m^3^ increase of PM_2.5_, PM_10_, SO_2_, NO_2_, and O_3_ in cancer-stratified analysis: Pancreas cancer.

| Lag days | PM_2.5_ (RR [95%CI]) |  | PM_10_ (RR [95%CI]) |  | SO_2_ (RR [95%CI]) |  | NO_2_ (RR [95%CI]) |  | O_3_ (RR [95%CI]) |
| --- | --- | --- | --- | --- | --- | --- | --- | --- | --- |
| lag 0 | 1.0020(0.9915,1.0126) |  | 1.0075(0.9996,1.0154) |  | 1.0114(0.9617,1.0636) |  | 0.9655(0.8133,1.1461) |  | 1.0060(0.9986,1.0135) |
| lag 1 | 1.0024(0.9937,1.0111) |  | 1.0063(0.9999,1.0126) |  | 1.0125(0.9725,1.0541) |  | 0.9818(0.8484,1.1363) |  | 1.0049(0.9989,1.0110) |
| lag 2 | 1.0027(0.9956,1.0098) |  | 1.0051(1.0000,1.0102) |  | 1.0136(0.9822,1.0460) |  | 0.9985(0.8800,1.1329) |  | 1.0039(0.9990,1.0088) |
| lag 3 | 1.0030(0.9968,1.0092) |  | 1.0039(0.9996,1.0081) |  | 1.0147(0.9895,1.0405) |  | 1.0154(0.9050,1.1392) |  | 1.0028(0.9986,1.0071) |
| lag 4 | 1.0033(0.9972,1.0095) |  | 1.0027(0.9985,1.0068) |  | 1.0158(0.9923,1.0398) |  | 1.0326(0.9205,1.1584) |  | 1.0018(0.9975,1.0060) |
| lag 5 | 1.0037(0.9967,1.0107) |  | 1.0015(0.9967,1.0063) |  | 1.0169(0.9896,1.0450) |  | 1.0501(0.9259,1.1910) |  | 1.0007(0.9958,1.0056) |
| lag 6 | 1.0040(0.9955,1.0126) |  | 1.0003(0.9943,1.0062) |  | 1.0180(0.9832,1.0541) |  | 1.0679(0.9233,1.2352) |  | 0.9996(0.9937,1.0057) |
| lag 7 | 1.0043(0.9940,1.0148) |  | 0.9991(0.9917,1.0065) |  | 1.0191(0.9750,1.0652) |  | 1.0860(0.9155,1.2883) |  | 0.9986(0.9912,1.0060) |
| lag 01 | 1.0044(0.9853,1.0238) |  | 1.0138(0.9996,1.0281) |  | 1.0240(0.9354,1.1210) |  | 0.9479(0.6907,1.3009) |  | 1.0110(0.9975,1.0246) |
| lag 02 | 1.0071(0.9812,1.0336) |  | 1.0189(0.9998,1.0384) |  | 1.0379(0.9197,1.1714) |  | 0.9465(0.6106,1.4672) |  | 1.0149(0.9967,1.0334) |
| lag 03 | 1.0101(0.9790,1.0422) |  | 1.0228(1.0000,1.0462) |  | 1.0532(0.9131,1.2147) |  | 0.9611(0.5593,1.6515) |  | 1.0178(0.9959,1.0401) |
| lag 04 | 1.0135(0.9781,1.0502) |  | 1.0255(0.9999,1.0518) |  | 1.0698(0.9141,1.2520) |  | 0.9924(0.5277,1.8663) |  | 1.0196(0.9948,1.0449) |
| lag 05 | 1.0172(0.9780,1.0579) |  | 1.0270(0.9992,1.0557) |  | 1.0879(0.9205,1.2856) |  | 1.0422(0.5090,2.1337) |  | 1.0203(0.9931,1.0482) |
| lag 06 | 1.0213(0.9781,1.0663) |  | 1.0273(0.9972,1.0583) |  | 1.1074(0.9290,1.3201) |  | 1.1130(0.4974,2.4904) |  | 1.0199(0.9901,1.0506) |
| lag 07 | 1.0257(0.9774,1.0763) |  | 1.0264(0.9933,1.0606) |  | 1.1286(0.9349,1.3624) |  | 1.2087(0.4874,2.9973) |  | 1.0185(0.9853,1.0528) |

Note: The bold number indicates the *P*<0.05.

RR, relative ratio; CI, confidence interval.

NO_2_, nitrogen dioxide; O_3_, ozone; PM_2.5_, particulate matter with an aerodynamic diameter <2.5 mm; PM_10_, particulate matter with an aerodynamic diameter <10 mm; SO_2_, sulfur dioxide.

Table S11. The lag-specific RRs and 95% CIs of GI cancer deaths associated with a 10μg/m^3^ increase of PM_2.5_, PM_10_, SO_2_, NO_2_, and O_3_ according to single-pollutant model: 6 df per year for time variable.

| Lag days | PM_2.5_ (RR [95%CI]) |  | PM_10_ (RR [95%CI]) |  | SO_2_ (RR [95%CI]) |  | NO_2_ (RR [95%CI]) |  | O_3_ (RR [95%CI]) |
| --- | --- | --- | --- | --- | --- | --- | --- | --- | --- |
| lag 0 | **1.0033(1.0004,1.0063)** |  | **1.0032(1.0010,1.0053)** |  | 1.0022(0.9899,1.0147) |  | 0.9881(0.9391,1.0396) |  | **1.0022(1.0001,1.0044)** |
| lag 1 | **1.0028(1.0004,1.0052)** |  | **1.0026(1.0008,1.0043)** |  | 1.0023(0.9924,1.0123) |  | 0.9959(0.9545,1.0390) |  | **1.0019(1.0001,1.0036)** |
| lag 2 | **1.0023(1.0003,1.0042)** |  | **1.0020(1.0006,1.0034)** |  | 1.0024(0.9946,1.0102) |  | 1.0037(0.9685,1.0402) |  | **1.0015(1.0001,1.0029)** |
| lag 3 | **1.0017(1.0001,1.0034)** |  | **1.0014(1.0002,1.0026)** |  | 1.0025(0.9961,1.0088) |  | 1.0116(0.9800,1.0443) |  | 1.0011(0.9999,1.0023) |
| lag 4 | 1.0012(0.9995,1.0029) |  | 1.0008(0.9997,1.0020) |  | 1.0026(0.9965,1.0087) |  | 1.0196(0.9878,1.0525) |  | 1.0007(0.9995,1.0019) |
| lag 5 | 1.0007(0.9988,1.0026) |  | 1.0002(0.9989,1.0016) |  | 1.0026(0.9954,1.0099) |  | 1.0276(0.9918,1.0648) |  | 1.0004(0.9990,1.0018) |
| lag 6 | 1.0002(0.9978,1.0025) |  | 0.9996(0.9979,1.0013) |  | 1.0027(0.9936,1.0120) |  | 1.0357(0.9930,1.0803) |  | 1.0000(0.9983,1.0017) |
| lag 7 | 0.9996(0.9967,1.0025) |  | 0.9990(0.9969,1.0011) |  | 1.0028(0.9913,1.0145) |  | 1.0439(0.9925,1.0980) |  | 0.9996(0.9975,1.0018) |
| lag 01 | **1.0061(1.0008,1.0115)** |  | **1.0058(1.0019,1.0097)** |  | 1.0045(0.9824,1.0272) |  | 0.9840(0.8967,1.0799) |  | **1.0041(1.0002,1.0080)** |
| lag 02 | **1.0084(1.0012,1.0156)** |  | **1.0078(1.0025,1.0130)** |  | 1.0069(0.9774,1.0374) |  | 0.9877(0.8697,1.1217) |  | **1.0056(1.0004,1.0109)** |
| lag 03 | **1.0102(1.0015,1.0189)** |  | **1.0092(1.0030,1.0155)** |  | 1.0094(0.9746,1.0456) |  | 0.9992(0.8557,1.1669) |  | **1.0067(1.0005,1.0130)** |
| lag 04 | **1.0114(1.0016,1.0213)** |  | **1.0100(1.0031,1.0170)** |  | 1.0120(0.9734,1.0521) |  | 1.0188(0.8523,1.2179) |  | **1.0075(1.0004,1.0146)** |
| lag 05 | **1.0121(1.0013,1.0230)** |  | **1.0102(1.0027,1.0179)** |  | 1.0147(0.9733,1.0578) |  | 1.0470(0.8573,1.2786) |  | **1.0078(1.0001,1.0156)** |
| lag 06 | **1.0122(1.0004,1.0242)** |  | **1.0099(1.0017,1.0181)** |  | 1.0175(0.9733,1.0636) |  | 1.0844(0.8681,1.3545) |  | 1.0078(0.9994,1.0163) |
| lag 07 | 1.0118(0.9986,1.0252) |  | 1.0089(0.9998,1.0180) |  | 1.0203(0.9722,1.0708) |  | 1.1320(0.8818,1.4532) |  | 1.0074(0.9980,1.0169) |

Note: The bold number indicates the *P*<0.05.

RR, relative ratio; CI, confidence interval.

NO_2_, nitrogen dioxide; O_3_, ozone; PM_2.5_, particulate matter with an aerodynamic diameter <2.5 mm; PM_10_, particulate matter with an aerodynamic diameter <10 mm; SO_2_, sulfur dioxide.

Table S12. The lag-specific RRs and 95% CIs of GI cancer deaths associated with a 10μg/m^3^ increase of PM_2.5_, PM_10_, SO_2_, NO_2_, and O_3_ according to single-pollutant model: 8 df per year for time variable.

| Lag days | PM_2.5_ (RR [95%CI]) |  | PM_10_ (RR [95%CI]) |  | SO_2_ (RR [95%CI]) |  | NO_2_ (RR [95%CI]) |  | O_3_ (RR [95%CI]) |
| --- | --- | --- | --- | --- | --- | --- | --- | --- | --- |
| lag 0 | **1.0033(1.0003,1.0063)** |  | **1.0030(1.0008,1.0052)** |  | 1.0031(0.9905,1.0159) |  | 0.9878(0.9383,1.0399) |  | **1.0023(1.0001,1.0044)** |
| lag 1 | **1.0028(1.0004,1.0053)** |  | **1.0024(1.0007,1.0042)** |  | 1.0030(0.9928,1.0133) |  | 0.9950(0.9530,1.0389) |  | **1.0019(1.0001,1.0036)** |
| lag 2 | **1.0023(1.0003,1.0044)** |  | **1.0019(1.0005,1.0033)** |  | 1.0028(0.9947,1.0109) |  | 1.0022(0.9662,1.0396) |  | **1.0015(1.0001,1.0029)** |
| lag 3 | **1.0018(1.0001,1.0036)** |  | **1.0014(1.0001,1.0026)** |  | 1.0026(0.9960,1.0093) |  | 1.0095(0.9768,1.0433) |  | 1.0011(0.9999,1.0024) |
| lag 4 | 1.0014(0.9996,1.0031) |  | 1.0008(0.9996,1.0020) |  | 1.0025(0.9961,1.0089) |  | 1.0169(0.9839,1.0510) |  | 1.0007(0.9995,1.0020) |
| lag 5 | 1.0009(0.9988,1.0029) |  | 1.0003(0.9989,1.0017) |  | 1.0023(0.9949,1.0098) |  | 1.0243(0.9872,1.0627) |  | 1.0003(0.9989,1.0018) |
| lag 6 | 1.0004(0.9979,1.0028) |  | 0.9997(0.9980,1.0015) |  | 1.0021(0.9928,1.0115) |  | 1.0317(0.9879,1.0775) |  | 1.0000(0.9982,1.0017) |
| lag 7 | 0.9999(0.9969,1.0028) |  | 0.9992(0.9971,1.0013) |  | 1.0020(0.9903,1.0138) |  | 1.0392(0.9869,1.0944) |  | 0.9996(0.9974,1.0018) |
| lag 01 | **1.0062(1.0007,1.0116)** |  | **1.0054(1.0015,1.0094)** |  | 1.0061(0.9834,1.0293) |  | 0.9828(0.8945,1.0800) |  | **1.0041(1.0002,1.0081)** |
| lag 02 | **1.0085(1.0011,1.0160)** |  | **1.0074(1.0020,1.0128)** |  | 1.0089(0.9785,1.0403) |  | 0.9850(0.8654,1.1212) |  | **1.0056(1.0003,1.0110)** |
| lag 03 | **1.0104(1.0014,1.0195)** |  | **1.0087(1.0023,1.0152)** |  | 1.0116(0.9755,1.0490) |  | 0.9944(0.8487,1.1652) |  | **1.0067(1.0004,1.0132)** |
| lag 04 | **1.0117(1.0015,1.0221)** |  | **1.0096(1.0023,1.0168)** |  | 1.0141(0.9739,1.0559) |  | 1.0112(0.8419,1.2145) |  | **1.0075(1.0003,1.0147)** |
| lag 05 | **1.0126(1.0012,1.0241)** |  | **1.0098(1.0019,1.0178)** |  | 1.0164(0.9732,1.0615) |  | 1.0358(0.8427,1.2730) |  | 1.0078(0.9999,1.0158) |
| lag 06 | **1.0130(1.0004,1.0257)** |  | **1.0096(1.0010,1.0182)** |  | 1.0186(0.9723,1.0670) |  | 1.0686(0.8485,1.3458) |  | 1.0078(0.9991,1.0166) |
| lag 07 | 1.0129(0.9988,1.0271) |  | 1.0088(0.9993,1.0184) |  | 1.0206(0.9701,1.0737) |  | 1.1105(0.8565,1.4400) |  | 1.0074(0.9976,1.0172) |

Note: The bold number indicates the *P*<0.05.

RR, relative ratio; CI, confidence interval.

NO_2_, nitrogen dioxide; O_3_, ozone; PM_2.5_, particulate matter with an aerodynamic diameter <2.5 mm; PM_10_, particulate matter with an aerodynamic diameter <10 mm; SO_2_, sulfur dioxide.

Table S13. The lag-specific RRs and 95% CIs of GI cancer deaths associated witha 10μg/m^3^ increase of PM_2.5_, PM_10_, SO_2_, NO_2_, and O_3_ according to single-pollutant model: 9 df per year for time variable.

| Lag days | PM_2.5_ (RR [95%CI]) |  | PM_10_ (RR [95%CI]) |  | SO_2_ (RR [95%CI]) |  | NO_2_ (RR [95%CI]) |  | O_3_ (RR [95%CI]) |
| --- | --- | --- | --- | --- | --- | --- | --- | --- | --- |
| lag 0 | **1.0032(1.0002,1.0062)** |  | **1.0031(1.0009,1.0053)** |  | 1.0040(0.9912,1.0170) |  | 0.9844(0.9348,1.0366) |  | **1.0024(1.0002,1.0046)** |
| lag 1 | **1.0028(1.0003,1.0053)** |  | **1.0025(1.0007,1.0043)** |  | 1.0038(0.9934,1.0143) |  | 0.9913(0.9492,1.0354) |  | **1.0019(1.0002,1.0037)** |
| lag 2 | **1.0023(1.0003,1.0044)** |  | **1.0020(1.0005,1.0034)** |  | 1.0036(0.9953,1.0119) |  | 0.9984(0.9621,1.0360) |  | **1.0015(1.0000,1.0030)** |
| lag 3 | **1.0019(1.0001,1.0037)** |  | **1.0014(1.0002,1.0027)** |  | 1.0034(0.9965,1.0103) |  | 1.0054(0.9724,1.0395) |  | 1.0011(0.9998,1.0023) |
| lag 4 | 1.0014(0.9996,1.0032) |  | 1.0009(0.9997,1.0021) |  | 1.0032(0.9966,1.0098) |  | 1.0125(0.9792,1.0470) |  | 1.0006(0.9994,1.0019) |
| lag 5 | 1.0009(0.9989,1.0030) |  | 1.0003(0.9989,1.0017) |  | 1.0030(0.9954,1.0106) |  | 1.0197(0.9824,1.0585) |  | 1.0002(0.9987,1.0016) |
| lag 6 | 1.0005(0.9980,1.0029) |  | 0.9998(0.9981,1.0015) |  | 1.0028(0.9933,1.0123) |  | 1.0269(0.9828,1.0730) |  | 0.9998(0.9980,1.0015) |
| lag 7 | 1.0000(0.9971,1.0030) |  | 0.9992(0.9971,1.0014) |  | 1.0026(0.9907,1.0145) |  | 1.0342(0.9816,1.0896) |  | 0.9993(0.9971,1.0015) |
| lag 01 | **1.0060(1.0005,1.0115)** |  | **1.0056(1.0016,1.0096)** |  | 1.0078(0.9847,1.0314) |  | 0.9759(0.8875,1.0730) |  | **1.0043(1.0004,1.0083)** |
| lag 02 | **1.0083(1.0009,1.0159)** |  | **1.0076(1.0022,1.0130)** |  | 1.0114(0.9804,1.0434) |  | 0.9743(0.8551,1.1100) |  | **1.0058(1.0005,1.0112)** |
| lag 03 | **1.0102(1.0012,1.0193)** |  | **1.0090(1.0026,1.0155)** |  | 1.0148(0.9779,1.0532) |  | 0.9795(0.8348,1.1493) |  | **1.0069(1.0005,1.0134)** |
| lag 04 | **1.0116(1.0013,1.0220)** |  | **1.0099(1.0026,1.0173)** |  | 1.0181(0.9768,1.0611) |  | 0.9918(0.8242,1.1934) |  | **1.0075(1.0002,1.0149)** |
| lag 05 | **1.0126(1.0011,1.0241)** |  | **1.0103(1.0023,1.0183)** |  | 1.0211(0.9764,1.0678) |  | 1.0113(0.8209,1.2459) |  | 1.0077(0.9997,1.0158) |
| lag 06 | **1.0130(1.0004,1.0258)** |  | **1.0100(1.0014,1.0188)** |  | 1.0239(0.9759,1.0742) |  | 1.0385(0.8222,1.3118) |  | 1.0075(0.9987,1.0164) |
| lag 07 | 1.0131(0.9989,1.0274) |  | 1.0093(0.9997,1.0190) |  | 1.0265(0.9740,1.0818) |  | 1.0740(0.8253,1.3977) |  | 1.0068(0.9969,1.0168) |

Note: The bold number indicates the P<0.05.

RR, relative ratio; CI, confidence interval.

NO_2_, nitrogen dioxide; O_3_, ozone; PM_2.5_, particulate matter with an aerodynamic diameter <2.5 mm; PM_10_, particulate matter with an aerodynamic diameter <10 mm; SO_2_, sulfur dioxide.

Table S14. The lag-specific RRs and 95% CIs of GI cancer deaths associated with a 10μg/m^3^ increase of PM_2.5_, PM_10_, SO_2_, NO_2_, and O_3_ according to single-pollutant model: 10 df per year for time variable.

| Lag days | PM_2.5_ (RR [95%CI]) |  | PM_10_ (RR [95%CI]) |  | SO_2_ (RR [95%CI]) |  | NO_2_ (RR [95%CI]) |  | O_3_ (RR [95%CI]) |
| --- | --- | --- | --- | --- | --- | --- | --- | --- | --- |
| lag 0 | 1.0029(0.9998,1.0059) |  | **1.0031(1.0009,1.0054)** |  | 1.0030(0.9901,1.0161) |  | 0.9893(0.9392,1.0422) |  | **1.0024(1.0002,1.0046)** |
| lag 1 | 1.0024(0.9999,1.0049) |  | **1.0026(1.0008,1.0044)** |  | 1.0027(0.9923,1.0133) |  | 0.9964(0.9536,1.0410) |  | **1.0020(1.0001,1.0038)** |
| lag 2 | 1.0019(0.9998,1.0040) |  | **1.0021(1.0006,1.0035)** |  | 1.0024(0.9941,1.0109) |  | 1.0034(0.9666,1.0417) |  | **1.0015(1.0000,1.0030)** |
| lag 3 | 1.0015(0.9996,1.0033) |  | **1.0015(1.0002,1.0028)** |  | 1.0021(0.9952,1.0092) |  | 1.0105(0.9769,1.0453) |  | 1.0011(0.9998,1.0024) |
| lag 4 | 1.0010(0.9992,1.0028) |  | 1.0010(0.9997,1.0022) |  | 1.0018(0.9951,1.0086) |  | 1.0177(0.9837,1.0528) |  | 1.0007(0.9994,1.0020) |
| lag 5 | 1.0005(0.9985,1.0026) |  | 1.0004(0.9990,1.0019) |  | 1.0015(0.9938,1.0093) |  | 1.0249(0.9870,1.0643) |  | 1.0003(0.9988,1.0018) |
| lag 6 | 1.0001(0.9976,1.0025) |  | 0.9999(0.9981,1.0017) |  | 1.0012(0.9917,1.0109) |  | 1.0322(0.9875,1.0789) |  | 0.9999(0.9981,1.0017) |
| lag 7 | 0.9996(0.9966,1.0026) |  | 0.9994(0.9972,1.0015) |  | 1.0009(0.9891,1.0129) |  | 1.0395(0.9862,1.0956) |  | 0.9995(0.9973,1.0017) |
| lag 01 | 1.0053(0.9998,1.0109) |  | **1.0057(1.0017,1.0098)** |  | 1.0058(0.9825,1.0296) |  | 0.9857(0.8959,1.0846) |  | **1.0043(1.0003,1.0084)** |
| lag 02 | 1.0072(0.9997,1.0149) |  | **1.0078(1.0023,1.0133)** |  | 1.0082(0.9770,1.0404) |  | 0.9891(0.8672,1.1282) |  | **1.0059(1.0004,1.0114)** |
| lag 03 | 1.0087(0.9995,1.0180) |  | **1.0093(1.0027,1.0159)** |  | 1.0104(0.9731,1.0490) |  | 0.9995(0.8505,1.1746) |  | **1.0070(1.0004,1.0136)** |
| lag 04 | 1.0097(0.9992,1.0203) |  | **1.0103(1.0029,1.0178)** |  | 1.0122(0.9706,1.0556) |  | 1.0172(0.8436,1.2265) |  | **1.0077(1.0002,1.0153)** |
| lag 05 | 1.0102(0.9986,1.0221) |  | **1.0107(1.0026,1.0190)** |  | 1.0138(0.9687,1.0609) |  | 1.0425(0.8441,1.2876) |  | 1.0080(0.9997,1.0164) |
| lag 06 | 1.0103(0.9974,1.0234) |  | **1.0106(1.0017,1.0196)** |  | 1.0150(0.9666,1.0659) |  | 1.0761(0.8493,1.3633) |  | 1.0079(0.9987,1.0172) |
| lag 07 | 1.0099(0.9954,1.0245) |  | **1.0100(1.0001,1.0200)** |  | 1.0159(0.9630,1.0717) |  | 1.1185(0.8565,1.4608) |  | 1.0074(0.9971,1.0178) |

Note: The bold number indicates the P<0.05.

RR, relative ratio; CI, confidence interval.

NO_2_, nitrogen dioxide; O_3_, ozone; PM_2.5_, particulate matter with an aerodynamic diameter <2.5 mm; PM_10_, particulate matter with an aerodynamic diameter <10 mm; SO_2_, sulfur dioxide.

Table S15. Table2.The lag-specific RRs and 95% CIs of GI cancer deaths associated with a 10μg/m^3^ increase of PM_2.5_, PM_10_, SO_2_, NO_2_, and O_3_ according to two-pollutant model.

| Two-pollutants models | Lag 0 day | Lag 1 day | Lag 01 day | Lag 02 day |
| --- | --- | --- | --- | --- |
| PM_2.5_ | **1.0031(1.0001,1.0061)** | **1.0026(1.0002,1.0050)** | **1.0057(1.0003,1.0111)** | **1.0078(1.0005,1.0152)** |
| PM_2.5_+O_3_ | 1.0017(0.9986,1.0047) | 1.0015(0.9990,1.0040) | 1.0031(0.9976,1.0086) | 1.0044(0.9970,1.0119) |
| PM_2.5_+NO_2_ | 1.0015(0.9984,1.0047) | 1.0014(0.9988,1.0040) | 1.0029(0.9972,1.0086) | 1.0041(0.9964,1.0119) |
| PM_2.5_+SO_2_ | 1.0020(0.9989,1.0052) | 1.0018(0.9992,1.0043) | 1.0038(0.9981,1.0095) | 1.0053(0.9976,1.0130) |
|  |  |  |  |  |
| PM_10_ | **1.0029(1.0007,1.0051)** | **1.0023(1.0006,1.0041)** | **1.0052(1.0013,1.0092)** | **1.0071(1.0017,1.0124)** |
| PM_10_+O_3_ | 1.0018(0.9995,1.0040) | 1.0015(0.9996,1.0033) | 1.0032(0.9992,1.0073) | 1.0044(0.9989,1.0099) |
| PM_10_+NO_2_ | 1.0017(0.9994,1.0041) | 1.0014(0.9996,1.0033) | 1.0032(0.9990,1.0074) | 1.0043(0.9987,1.0100) |
| PM_10_+SO_2_ | 1.0021(0.9998,1.0045) | 1.0018(0.9999,1.0036) | 1.0039(0.9997,1.0081) | 1.0053(0.9997,1.0109) |
|  |  |  |  |  |
| SO_2_ | 1.0026(0.9901,1.0152) | 1.0026(0.9926,1.0127) | 1.0052(0.9827,1.0281) | 1.0078(0.9779,1.0387) |
| SO_2_+NO_2_ | 1.0027(0.9901,1.0155) | 1.0027(0.9926,1.0129) | 1.0054(0.9828,1.0286) | 1.0081(0.9778,1.0393) |
| SO_2_+O_3_ | 1.0033(0.9909,1.0160) | 1.0032(0.9932,1.0133) | 1.0065(0.9842,1.0294) | 1.0096(0.9797,1.0405) |
| SO_2_+PM_2.5_ | 1.0038(0.9906,1.0170) | 1.0035(0.9930,1.0141) | 1.0073(0.9837,1.0313) | 1.0105(0.9791,1.0429) |
| SO_2_+PM_10_ | 1.0046(0.9915,1.0180) | 1.0042(0.9937,1.0148) | 1.0088(0.9852,1.0330) | 1.0126(0.9811,1.0452) |
|  |  |  |  |  |
| NO_2_ | 0.9877(0.9384,1.0395) | 0.9950(0.9533,1.0385) | 0.9828(0.8949,1.0792) | 0.9851(0.8664,1.1201) |
| NO_2_+O_3_ | 0.9878(0.9385,1.0396) | 0.9953(0.9536,1.0388) | 0.9831(0.8953,1.0796) | 0.9859(0.8672,1.1210) |
| NO_2_+SO_2_ | 0.9871(0.9378,1.0389) | 0.9944(0.9527,1.0379) | 0.9815(0.8937,1.0779) | 0.9832(0.8646,1.1180) |
| NO_2_+PM_2.5_ | 0.9872(0.9379,1.0391) | 0.9946(0.9529,1.0382) | 0.9819(0.8940,1.0784) | 0.9840(0.8653,1.1189) |
| NO_2_+PM_10_ | 0.9872(0.9379,1.0392) | 0.9946(0.9529,1.0382) | 0.9819(0.8940,1.0785) | 0.9839(0.8651,1.1190) |
|  |  |  |  |  |
| O_3_ | **1.0024(1.0002,1.0045)** | **1.0020(1.0002,1.0037)** | **1.0044(1.0004,1.0083)** | **1.0060(1.0007,1.0113)** |
| O_3_+NO_2_ | 1.0017(0.9999,1.0035) | 1.0014(1.0000,1.0028) | 1.0037(0.9997,1.0077) | 1.0051(0.9997,1.0105) |
| O_3_+SO_2_ | **1.0023(1.0001,1.0045)** | **1.0019(1.0001,1.0037)** | **1.0042(1.0002,1.0081)** | **1.0057(1.0004,1.0111)** |
| O_3_+PM_2.5_ | 1.0021(0.9999,1.0043) | 1.0018(1.0000,1.0036) | 1.0039(0.9998,1.0079) | 1.0053(0.9999,1.0108) |
| O_3_+PM_10_ | 1.0021(0.9999,1.0043) | 1.0018(1.0000,1.0036) | 1.0039(0.9999,1.0079) | 1.0053(0.9999,1.0108) |

Note: The bold number indicates the *P*<0.05.

RR, relative ratio; CI, confidence interval.

NO_2_, nitrogen dioxide; O_3_, ozone; PM_2.5_, particulate matter with an aerodynamic diameter <2.5 mm; PM_10_, particulate matter with an aerodynamic diameter <10 mm; SO_2_, sulfur dioxide.

TableS16. The lag-specific RRs and 95% CIs of GI cancer deaths associated with a 10μg/m^3^ increase of PM_2.5_, PM_10_, SO_2_, NO_2_, and O_3_ with dummy variables of the SARS-CoV-2 pandemic.

| Lag days | PM_2.5_ (RR [95%CI]) |  | PM_10_ (RR [95%CI]) |  | SO_2_ (RR [95%CI]) |  | NO_2_ (RR [95%CI]) |  | O_3_ (RR [95%CI]) |
| --- | --- | --- | --- | --- | --- | --- | --- | --- | --- |
| lag 0 | **1.0031(1.0001,1.0061)** |  | **1.0029(1.0007,1.0051)** |  | 1.0026(0.9901,1.0152) |  | 0.9877(0.9384,1.0395) |  | **1.0024(1.0002,1.0045)** |
| lag 1 | **1.0026(1.0002,1.0050)** |  | **1.0023(1.0006,1.0041)** |  | 1.0026(0.9926,1.0127) |  | 0.9950(0.9533,1.0385) |  | **1.0020(1.0002,1.0037)** |
| lag 2 | **1.0021(1.0001,1.0041)** |  | **1.0018(1.0004,1.0032)** |  | 1.0026(0.9947,1.0106) |  | 1.0024(0.9668,1.0394) |  | **1.0016(1.0002,1.0030)** |
| lag 3 | 1.0016(0.9999,1.0034) |  | **1.0013(1.0001,1.0025)** |  | 1.0027(0.9962,1.0092) |  | 1.0099(0.9777,1.0431) |  | 1.0012(1.0000,1.0024) |
| lag 4 | 1.0011(0.9994,1.0028) |  | 1.0007(0.9995,1.0019) |  | 1.0027(0.9965,1.0090) |  | 1.0174(0.9849,1.0509) |  | 1.0008(0.9996,1.0020) |
| lag 5 | 1.0006(0.9987,1.0026) |  | 1.0002(0.9988,1.0016) |  | 1.0028(0.9954,1.0102) |  | 1.0249(0.9884,1.0627) |  | 1.0004(0.9990,1.0018) |
| lag 6 | 1.0001(0.9977,1.0025) |  | 0.9997(0.9979,1.0014) |  | 1.0028(0.9935,1.0122) |  | 1.0325(0.9892,1.0777) |  | 1.0000(0.9983,1.0018) |
| lag 7 | 0.9996(0.9967,1.0026) |  | 0.9991(0.9970,1.0012) |  | 1.0029(0.9912,1.0147) |  | 1.0402(0.9883,1.0948) |  | 0.9996(0.9975,1.0018) |
| lag 01 | **1.0057(1.0003,1.0111)** |  | **1.0052(1.0013,1.0092)** |  | 1.0052(0.9827,1.0281) |  | 0.9828(0.8949,1.0792) |  | **1.0044(1.0004,1.0083)** |
| lag 02 | **1.0078(1.0005,1.0152)** |  | **1.0071(1.0017,1.0124)** |  | 1.0078(0.9779,1.0387) |  | 0.9851(0.8664,1.1201) |  | **1.0060(1.0007,1.0113)** |
| lag 03 | **1.0094(1.0006,1.0184)** |  | **1.0083(1.0020,1.0147)** |  | 1.0105(0.9751,1.0473) |  | 0.9949(0.8504,1.1639) |  | **1.0072(1.0008,1.0135)** |
| lag 04 | **1.0105(1.0005,1.0207)** |  | **1.0091(1.0019,1.0163)** |  | 1.0133(0.9739,1.0543) |  | 1.0121(0.8446,1.2129) |  | **1.0080(1.0008,1.0152)** |
| lag 05 | **1.0112(1.0001,1.0224)** |  | **1.0093(1.0015,1.0171)** |  | 1.0161(0.9738,1.0602) |  | 1.0373(0.8466,1.2711) |  | **1.0084(1.0005,1.0163)** |
| lag 06 | 1.0113(0.9991,1.0237) |  | **1.0089(1.0005,1.0174)** |  | 1.0190(0.9738,1.0663) |  | 1.0711(0.8538,1.3437) |  | 1.0084(0.9998,1.0170) |
| lag 07 | 1.0109(0.9973,1.0248) |  | 1.0080(0.9987,1.0174) |  | 1.0219(0.9725,1.0738) |  | 1.1141(0.8632,1.4380) |  | 1.0080(0.9984,1.0177) |

Note: The bold number indicates the *P*<0.05.

RR, relative ratio; CI, confidence interval.

NO_2_, nitrogen dioxide; O_3_, ozone; PM_2.5_, particulate matter with an aerodynamic diameter <2.5 mm; PM_10_, particulate matter with an aerodynamic diameter <10 mm; SO_2_, sulfur dioxide.

TableS17. Cumulative RRs and 95% CIs of GI cancer deaths associated with a 10μg/m³ increase in PM_2.5_, PM_10_, SO_2_, NO_2_, and O_3_ over 0–21 days (single-pollutant model).

| Lag days | PM_2.5_ (RR [95%CI] |  | PM_10_ (RR [95%CI]) |  | SO_2_ (RR [95%CI] |  | NO_2_ (RR [95%CI] |  | O_3_ (RR [95%CI]) |
| --- | --- | --- | --- | --- | --- | --- | --- | --- | --- |
| lag 0 | 1.0015(0.9994,1.0036) |  | 1.0010(0.9996,1.0025) |  | 1.0024(0.9957,1.0092) |  | 1.0202(0.9792,1.0628) |  | 1.0007(0.9992,1.0022) |
| lag 01 | 1.0030(0.9989,1.0071) |  | 1.0020(0.9993,1.0048) |  | 1.0049(0.9919,1.0180) |  | 1.0397(0.9603,1.1257) |  | 1.0014(0.9985,1.0042) |
| lag 02 | 1.0044(0.9984,1.0104) |  | 1.0030(0.9989,1.0070) |  | 1.0074(0.9884,1.0267) |  | 1.0586(0.9429,1.1885) |  | 1.0020(0.9978,1.0062) |
| lag 03 | 1.0057(0.9980,1.0135) |  | 1.0038(0.9986,1.0091) |  | 1.0099(0.9853,1.0350) |  | 1.0768(0.9271,1.2507) |  | 1.0027(0.9973,1.0081) |
| lag 04 | 1.0070(0.9977,1.0164) |  | 1.0046(0.9983,1.0110) |  | 1.0124(0.9826,1.0431) |  | 1.0943(0.9127,1.3120) |  | 1.0033(0.9967,1.0099) |
| lag 05 | 1.0082(0.9974,1.0192) |  | 1.0054(0.9980,1.0128) |  | 1.0150(0.9802,1.0510) |  | 1.1109(0.8994,1.3721) |  | 1.0040(0.9963,1.0117) |
| lag 06 | 1.0094(0.9971,1.0218) |  | 1.0061(0.9978,1.0145) |  | 1.0176(0.9781,1.0587) |  | 1.1267(0.8873,1.4307) |  | 1.0046(0.9959,1.0134) |
| lag 07 | 1.0105(0.9969,1.0243) |  | 1.0068(0.9975,1.0161) |  | 1.0202(0.9763,1.0661) |  | 1.1416(0.8761,1.4876) |  | 1.0052(0.9956,1.0150) |
| lag 08 | 1.0116(0.9967,1.0267) |  | 1.0074(0.9973,1.0175) |  | 1.0229(0.9747,1.0735) |  | 1.1556(0.8657,1.5426) |  | 1.0058(0.9953,1.0165) |
| lag 09 | 1.0126(0.9965,1.0289) |  | 1.0079(0.9970,1.0189) |  | 1.0256(0.9733,1.0806) |  | 1.1686(0.8560,1.5955) |  | 1.0065(0.9951,1.0180) |
| lag 10 | 1.0135(0.9964,1.0309) |  | 1.0084(0.9968,1.0201) |  | 1.0283(0.9721,1.0877) |  | 1.1807(0.8467,1.6463) |  | 1.0071(0.9949,1.0194) |
| lag 11 | 1.0144(0.9962,1.0329) |  | 1.0088(0.9965,1.0213) |  | 1.0311(0.9711,1.0947) |  | 1.1917(0.8378,1.6950) |  | 1.0077(0.9947,1.0208) |
| lag 12 | 1.0152(0.9960,1.0347) |  | 1.0092(0.9962,1.0224) |  | 1.0338(0.9702,1.1017) |  | 1.2016(0.8290,1.7416) |  | 1.0082(0.9945,1.0221) |
| lag 13 | 1.0160(0.9958,1.0365) |  | 1.0095(0.9959,1.0233) |  | 1.0367(0.9693,1.1087) |  | 1.2104(0.8202,1.7863) |  | 1.0088(0.9944,1.0235) |
| lag 14 | 1.0167(0.9956,1.0382) |  | 1.0098(0.9955,1.0242) |  | 1.0395(0.9685,1.1158) |  | 1.2181(0.8112,1.8293) |  | 1.0094(0.9943,1.0248) |
| lag 15 | 1.0173(0.9953,1.0398) |  | 1.0100(0.9951,1.0251) |  | 1.0424(0.9676,1.1230) |  | 1.2247(0.8017,1.8709) |  | 1.0100(0.9941,1.0261) |
| lag 16 | 1.0179(0.9949,1.0414) |  | 1.0101(0.9946,1.0259) |  | 1.0453(0.9666,1.1305) |  | 1.2301(0.7916,1.9116) |  | 1.0105(0.9939,1.0274) |
| lag 17 | 1.0184(0.9945,1.0429) |  | 1.0102(0.9941,1.0266) |  | 1.0483(0.9655,1.1381) |  | 1.2343(0.7806,1.9519) |  | 1.0111(0.9937,1.0287) |
| lag 18 | 1.0189(0.9939,1.0444) |  | 1.0103(0.9934,1.0274) |  | 1.0513(0.9643,1.1461) |  | 1.2374(0.7685,1.9922) |  | 1.0116(0.9935,1.0301) |
| lag 19 | 1.0193(0.9933,1.0460) |  | 1.0102(0.9927,1.0281) |  | 1.0543(0.9628,1.1544) |  | 1.2392(0.7553,2.0333) |  | 1.0121(0.9932,1.0315) |
| lag 20 | 1.0196(0.9924,1.0475) |  | 1.0102(0.9919,1.0288) |  | 1.0573(0.9611,1.1632) |  | 1.2399(0.7406,2.0758) |  | 1.0127(0.9928,1.0329) |
| lag 21 | 1.0199(0.9915,1.0491) |  | 1.0100(0.9909,1.0295) |  | 1.0604(0.9591,1.1725) |  | 1.2393(0.7243,2.1204) |  | 1.0132(0.9923,1.0344) |

Note: RR, relative ratio; CI, confidence interval.

NO_2_, nitrogen dioxide; O_3_, ozone; PM_2.5_, particulate matter with an aerodynamic diameter <2.5 mm; PM_10_, particulate matter with an aerodynamic diameter <10 mm; SO_2_, sulfur dioxide.
